# Trehalose-Functionalized Magnetic Affinity Probe Provides Biochemical Evidence of Nanoparticle Internalization in Mycobacteria

**DOI:** 10.1021/acsinfecdis.5c00506

**Published:** 2025-09-26

**Authors:** Harini A. Perera, N. G. Hasitha Raviranga, Olof Ramström, Mingdi Yan

**Affiliations:** † Department of Chemistry, University of Massachusetts Lowell, One University Avenue, Lowell, Massachusetts 01854, United States; ‡ Department of Chemistry and Biomedical Sciences, Linnaeus University, SE-39182 Kalmar, Sweden

**Keywords:** nanoparticles, internalization, photoaffinity labeling, Mycobacterium smegmatis, bacterial proteins, trehalose

## Abstract

We developed a magnetic affinity probe (MAP), consisting of iron oxide magnetic nanoparticles (MNP) functionalized with a photoaffinity labeling agent perfluorophenyl azide (PFPA), to characterize the internalization of nanoparticles by *Mycobacterium smegmatis*. Two MAPs were synthesized: a trehalose-functionalized MAP, PFPA-MNP-Tre, and an ethanol-functionalized MAP, PFPA-MNP-OH. Following incubation of MAP with bacteria, the samples were irradiated to trigger covalent bond formation between PFPA and bacterial proteins. The captured proteins were isolated by cleaving the disulfide bond in the linkers and removing the magnetic nanoparticles by using a magnet. For PFPA-MNP-Tre incubated with *M. smegmatis* for 24 h, proteomic analysis revealed that the captured proteins are cytoplasmic mycobacterial proteins, which provided biochemical evidence for the internalization of nanoparticles in bacteria. Additionally, PFPA-MNP-Tre accumulated at the poles of the mycobacteria, and the amount of captured proteins decreased with increasing concentration of added free trehalose. These results underscore the role the surface ligand plays in modulating the uptake of nanoparticles. The modular MAP platform may find broad applications in studying mechanisms and processes involving nanoparticle–cell interactions.

## Graphical abstract

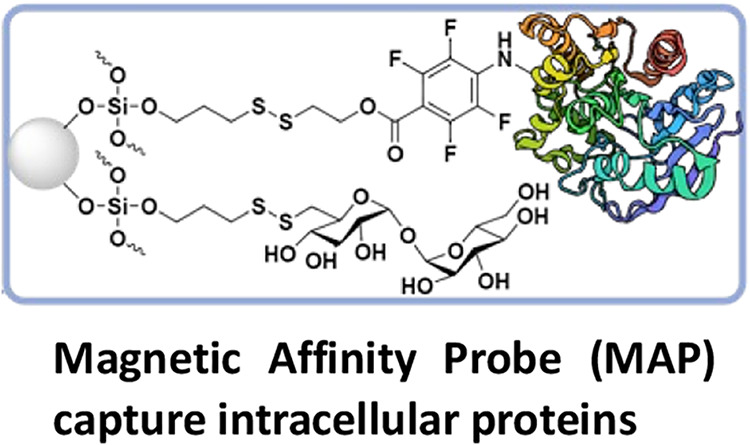

Antibiotics, which are often secondary metabolites of microorganisms, target biological processes that are important for the growth and survival of bacteria. However, bacteria have developed multiple mechanisms to render the traditional antibiotics ineffective, and as such, treating multidrug-resistant (MDR) bacteria would require antibiotics in high dosages and sometimes in combination with other antibiotics, which may cause unfavorable adverse effects or drug interactions.^1^ Nanotherapeutics, such as nanomaterials that are intrinsically antimicrobial or nanocarriers that can deliver antimicrobial compounds, have increasingly been investigated as alternatives to conventional antibiotics in the fight against MDR.^2,3^ Nanomaterials offer a number of potential advantages, including multivalent interactions, potentially favorable pharmacokinetics and biodistribution profiles, the ability to overcome bacteria cell wall barriers, and multiple modes of action (MOAs).^4,5^ A common MOA of nanotherapeutics is oxidative stress resulting from the interactions of nanoparticles with bacteria, leading to the production of reactive oxygen species and resulting in cellular damage, among others.^6^ Metal and metal oxide nanoparticles can release metal ions, which upon reaching intracellular compartments, interact with different functional groups such as amine, carboxyl and thiol on proteins and nucleic acids, and disturb enzymatic activities and physiological functions in bacteria.^7^ Other proposed mechanisms include accumulation of nanoparticles on the bacterial cell wall, causing cell wall perforation,^8^ and increased cell volume resulting in membrane leakage.^9^

For intracellular MOAs, the ability to cross the bacterial cell wall is the first critical step for nanotherapeutics to be effective.^10^ In mammalian cells, active ingestion mechanisms such as endocytosis are the primary pathways for the cellular uptake of nanoparticles.^11^ Cells process nanoparticles through various digestive enzymes in the lysosomes before eventually removing them.^12,13^ It is generally accepted that bacteria, with a few exceptions such as the Planctomycetes phylum that displays certain similarities to eukaryotic cells, such as compartmentalization,^14^ lack endocytosis mechanisms due to the rigidity of their cell walls, preventing particle internalization by this route.^15^ Gram-negative bacteria have a particularly impermeable outer membrane, which presents a formidable barrier for antibiotic uptake.^16^ This challenge is even greater in mycobacteria, whose cell envelope is estimated to be 100–1000 times less permeable.^17^

Various strategies have been developed to overcome this challenge. It has been shown that the uptake of nanoparticles into bacteria is significantly affected by the size of the nanoparticles and the surface ligand.^18^ For example, Sharma et al. has shown that for gold nanoparticles functionalized with positively charged cell penetrating peptides, 5 nm particles could attach to negatively charged bacterial surfaces, leading to cell membrane disruption, whereas 10 nm particles could not.^19^ Surface ligands act as the interface between nanoparticles and bacteria and play an important role in governing these interactions.^20^ As such, the chemical structure and charge state of the surface ligand can significantly impact the interactions of nanoparticles with bacteria.^21,22^ In certain applications, ligands are specifically introduced to the nanoparticle surface to enhance the activity of nanoparticles by promoting bacterial binding. For instance, positively charged ligands, such as cationic quaternary ammonium or chitosan, interact strongly with the negatively charged bacterial surface, leading to increased antimicrobial activity of nanoparticles by damaging the bacterial membrane.^23–25^ We found that bacterium-specific carbohydrates as the surface ligand can significantly enhance the interactions of nanoparticles with bacteria, increase nanoparticle uptake, and improve the antimicrobial activity of nanoparticles.^20,26,27^ For instance, the disaccharide trehalose is crucial for the survival and pathogenicity of mycobacteria but is absent in mammalian biology.^28^ Nanoparticles surface functionalized with trehalose interacted strongly with mycobacteria, whereas minimal interactions were observed for unfunctionalized nanoparticles or nanoparticles functionalized with other carbohydrates.^29–31^ Furthermore, trehalose as the surface ligand enhanced the antibacterial activities of antibiotic-loaded mesoporous silica nanoparticles^32,33^ and silver nanoparticles.^34^ This was attributed to increased intracellular concentrations of antibiotic or silver ions resulting from enhanced interactions of trehalose-functionalized nanoparticles with mycobacteria.^32^

Several techniques have been used to characterize the internalization of nanoparticles in bacteria. One such method involves embedding the nanoparticle-treated bacteria inside a solid matrix, followed by slicing the sample into thin sections and imaging under transmission electron microscopy (TEM).^35–37^ Observation of nanoparticles in the interior of the bacterial cells supports internalization. A drawback of this technique is that it is difficult to rule out the possibility of nanoparticle contamination when slicing the samples. For metal nanoparticles, inductively coupled plasma atomic emission spectroscopy (ICP-AES), inductively coupled plasma mass spectrometry (ICP-MS), and atomic absorption spectroscopy (AAS) have been used to quantify the concentration of metal ions in metal nanoparticle-treated bacteria.^22,38,39^ While these techniques are capable of measuring low concentrations of metal ions, they cannot differentiate between internalized and physically adsorbed nanoparticles. Xu and co-workers employed surface plasmon resonance (SPR) spectroscopy to differentiate internalized from surface-adhered silver nanoparticles.^40^ The surface adhered nanoparticles are brighter due to higher light scattering than intracellular nanoparticles, which are weaker. When nanoparticles are intrinsically fluorescent or carry a fluorescent payload, their interactions with bacteria can be studied by fluorescence microscopy or flow cytometry.^41–43^ However, it is difficult for fluorescence-based techniques to definitively distinguish between nanoparticles that are internalized and those that are adhered on a bacterial surface.

In this work, we developed a new method that provides direct biochemical evidence for the internalization of nanoparticles in bacteria by capturing cytoplasmic bacterial proteins by using a magnetic affinity probe (MAP). The MAP consists of iron oxide magnetic nanoparticle (MNP) functionalized with a binding ligand and a photoaffinity labeling agent, i.e., perfluorophenyl azide (PFPA) (Scheme 1A). PFPAs are classic photoaffinity labeling agents widely used to covalently capture proteins through photochemically initiated C–H insertion reactions (Scheme 1B).^44,45^ PFPA offers several advantages, including fast kinetics and high reaction yields, making it one of the most efficient photoaffinity labeling agents for studying protein–protein, lipid–protein, and carbohydrate–lectin interactions.^46,47^ Additionally, the photochemical reaction allows for spatiotemporal control, enabling the capture of bacterial proteins on demand. This feature is lacking in specific protein-capturing probes, such as *N*-hydroxysuccinimide ester, that do not offer spatiotemporal control.^48^

**Scheme 1:**
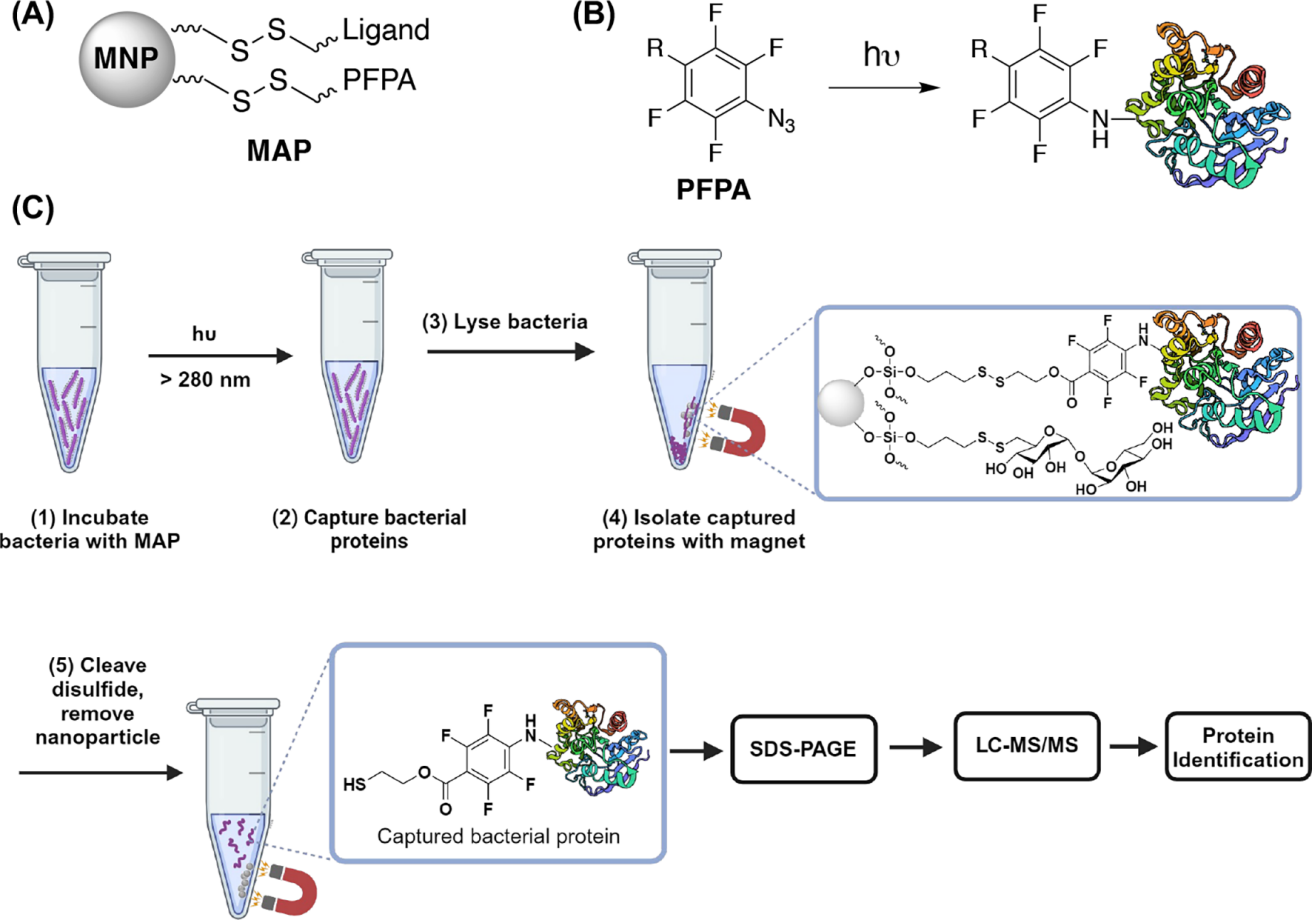
(A) General MAP Structure; (B) Photochemically Initiated C–H Insertion Reaction of PFPA with Protein; (C) Workflow of PFPA-MNP-Tre to Capture and Isolate Mycobacterial Proteins^a^

a The sizes of nanoparticle, ligands, and protein are not drawn to scale. For clarity, only one PFPA and one ligand is shown. (C) was created with BioRender.com.

While small-molecule photoaffinity probes are common, nanoparticle-based photoaffinity probes are scarce. The one designed by the Okada group, consisting of gold nanoparticles functionalized with β-d-lactose as the binding ligand and benzophenone as the photoaffinity labeling agent, was used to capture β-d-lactose-binding proteins.^49^ Mori and Sakurai designed a clickable nanoparticle probe to label oxysterol-binding protein in SNU-1 gastric carcinoma cell lysate.^50^ The probe consists of gold nanoparticles functionalized with the photoaffinity labeling agent diazirine and an azide through which the binding ligand, alkyne-tagged cholenic acid, was conjugated via the click reaction. Neither of these nanoparticle-based photoaffinity probes was used in live bacteria. The ability to capture bacterial proteins in live cells has not been demonstrated.

We hypothesize that if MAPs are internalized in bacteria through the photoaffinity labeling action of PFPA, we will be able to covalently capture cytoplasmic bacterial proteins, thus providing direct biochemical evidence to confirm the internalization of nanoparticles in bacteria. Using Mycobacterium as the model bacterium, we designed and synthesized two MAPs: a trehalose-functionalized MAP, PFPA-MNP-Tre, and an ethanol-functionalized MAP, PFPA-MNP-OH. MNPs were chosen owing to the straightforward preparation protocol, biocompatibility, and ease of separation by using a magnet.^51,52^ Both PFPA and the ligand contain a disulfide linker (Scheme 1C), which can be cleaved to release the captured proteins for subsequent separation and mass spectrometry analysis. The process of using MAP to capture and isolate bacterial proteins *in situ* consists of five main steps (Scheme 1): (1) Incubate MAP with bacteria, (2) irradiate to capture bacterial proteins through covalent bond formation with PFPA, (3) lyse bacteria, (4) isolate protein-MAP using a magnet, and (5) release captured proteins by cleaving the disulfide bond and remove nanoparticles. The isolated proteins are subjected to sodium dodecyl sulfate-polyacrylamide gel electrophoresis (SDS-PAGE) and protein bands are analyzed by liquid chromatography–tandem mass spectrometry (LC-MS/MS). Following this process, we have successfully isolated and captured proteins. For PFPA-MNP-Tre incubated with *Mycobacterium smegmatis* for 24 h, our results showed that PFPA-MNP-Tre captured cytoplasmic proteins.

## Results

### Synthesis of PFPA-MNP-Tre

PFPA-MNP-Tre and PFPA-MNP-OH were synthesized following the reaction sequence, according to Scheme 2. MNPs were synthesized by the thermal decomposition method from iron­(III) acetylacetonate in the presence of 1,2-hexadecanediol, oleic acid, and oleylamine (Scheme S1).^53^ The particles were characterized by Fourier transform infrared (FT-IR) spectroscopy (Figure S1A) and dynamic light scattering (DLS) which gave a hydrodynamic diameter of 6.0 ± 0.8 nm and polydispersity index of 0.66 ± 0.03 (Figure S1B and Table 1). The nanoparticles can be readily precipitated by using a magnet (Figure S1C).

**Scheme 2:**
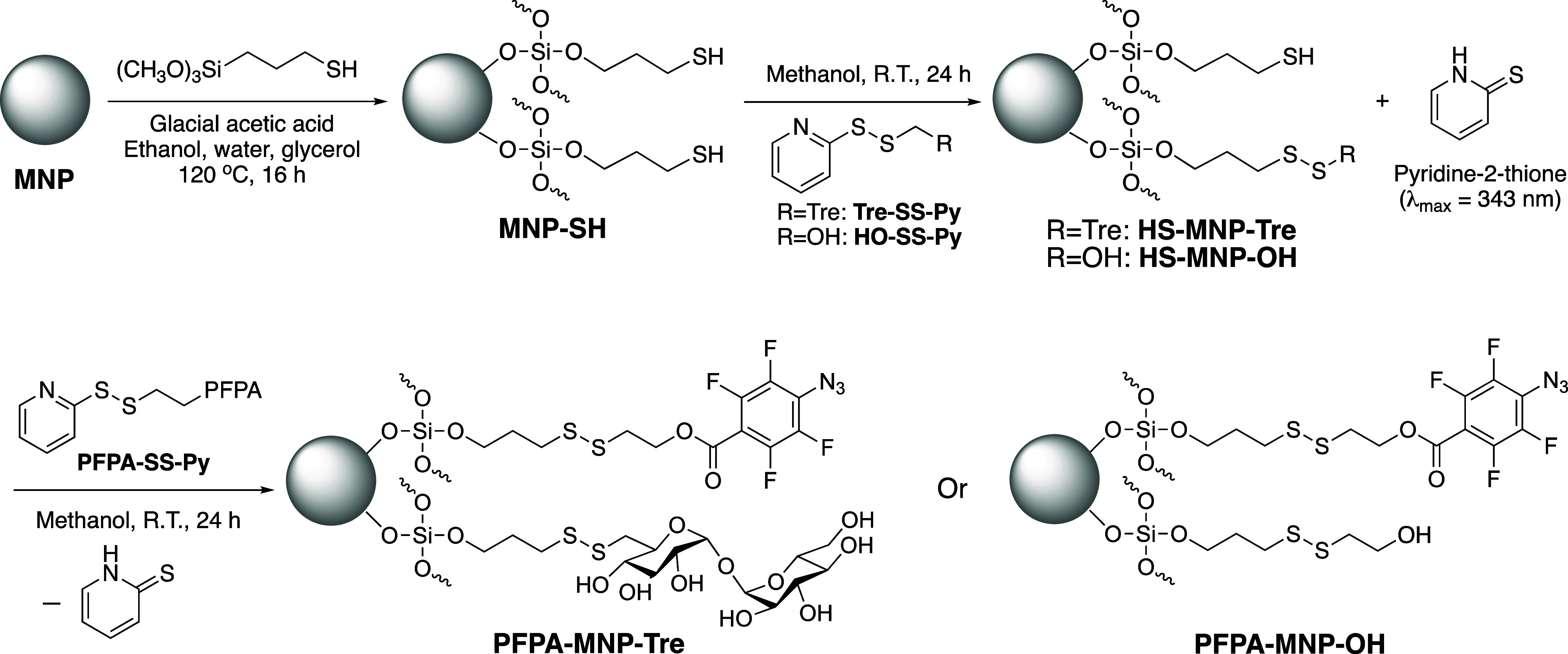
Synthesis of PFPA-MNP-Tre and PFPA-MNP-OH^a^

a RT: room temperature. Only one PFPA and one ligand each on the nanoparticle are shown for clarity. Synthesis and characterization data of Tre-SS-Py, HO-SS-Py, and PFPA-SS-Py can be found in Supporting Information (SI).

**Table 1:** Characterizations of Nanoparticles^a^

	diameter (nm)^b^	polydispersity index^b^	ζ potential (mV)^c^	[Thiol] (×10^4^ mmol/mg)^d^	conjugation yield (%)	
MNP	6.0 ± 0.8	0.66 ± 0.03	ND^e^	0.090 ± 0.008		
MNP-SH	6.1 ± 2.2	1.40 ± 0.92	–(84.8 ± 7.3)	1.86 ± 0.81		
PFPA-MNP-Tre	10.6 ± 0.6	0.4 ± 0.1	–(9.4 ± 1.0)	0.26 ± 0.11	74 ± 3^f^	85 ± 8^g^
PFPA-MNP-OH	8.8 ± 0.4	1.0 ± 0.3	–(3.3 ± 0.9)	0.11 ± 0.06	85 ± 2^f^	93 ± 6^h^

a Results are presented as average ± standard deviation.

b Measured by DLS. MNPs were dispersed in hexane and all other particles in water containing 10 mM KNO_3_.

c Measured in pH 7.4 phosphate-buffered saline (PBS) buffer. The ζ potential data of three samples each of PFPA-MNP-Tre and PFPA-MNP-OH can be found in Table S2.

d Measured by the Ellman assay. The thiol concentration of three samples each of MNP, MNP-SH, PFPA-MNP-Tre, and PFPA-MNP-OH can be found in Table S2.

e Not determined due to poor solubility of MNPs in PBS.

f Yield% calculated by measuring the amount of pyridine-2-thione released from the reaction. Yield% for each step and of three samples each of PFPA-MNP-Tre and PFPA-MNP-OH can be found in Table S1.

g Yield% = ([Thiol]_MNP‑SH_ – [Thiol]_PFPA‑MNP‑Tre_)/[Thiol]_MNP‑SH_ × 100%. Yield% of three samples each of PFPA-MNP-Tre and PFPA-MNP-OH can be found in Table S2.

h Yield% = ([Thiol]_MNP‑SH_ – [Thiol]_PFPA‑MNP‑OH_)/[Thiol]_MNP‑SH_ × 100%. Yield% of three samples each of PFPA-MNP-Tre and PFPA-MNP-OH can be found in Table S2.

MNPs were subsequently treated with (3-mercaptopropyl)­trimethoxysilane to give thiol-functionalized MNPs, MNP-SH (Scheme S2). The hydrodynamic diameter and polydispersity index of MNP-SH were measured to be 6.1 ± 2.2 and 1.40 ± 0.92, respectively (Figure S2A and Table 1), slightly less uniform than those of MNPs. The thiol concentration on MNP-SH was quantified using the Ellman assay.^54,55^ The assay involves the reduction of the sulfhydryl group by the Ellman reagent 5,5-dithio-bis­(2-nitrobenzoic acid) (DTNB) to give 2-nitro-5-thiobenzoic acid (TNB), which is a yellow-colored compound with a molar extinction coefficient of 13,600 M^–1^ cm^–1^ at 412 nm and pH 8.0.^56^ To determine the thiol concentration on MNP-SH, weighed samples were added to DTNB, where the color of the DTNB solution turned dark yellow (Figure S18). After the nanoparticles were removed by centrifugation, the absorbance of the supernatant at 412 nm was compared to a calibration (Figure S19) to determine the thiol concentration. Using this method, the thiol concentration on MNP-SH was calculated to be (1.86 ± 0.81) × 10^–4^ mmol/mg particle (Table 1).

In this study, the PFPA/ligand feed ratio was set at 1:1 to ensure sufficient PFPA for protein capture and to facilitate characterization of nanoparticle surface for both PFPA and the trehalose or ethanol ligand. PFPA-MNP-Tre was synthesized in two steps (Scheme 2) by first treating MNP-SH with pyridine disulfide-derivatized trehalose, Tre-SS-Py (Scheme S4), and then PFPA-SS-Py (Scheme S3A), each added at 0.5 equiv of the total thiol concentration on MNP-SH. Both reactions generated pyridine-2-thione as the byproduct, which is a bright yellow compound with λ_max_ at 343 nm. By measuring the absorbance of the reaction mixture at 343 nm, the reaction progress can be monitored. The reaction was deemed completed when the concentration of released pyridine-2-thione remained unchanged, which took about 24 h for both steps. At the completion of the reaction, the nanoparticles were removed by centrifugation, and the concentration of pyridine-2-thione in the supernatant was quantified against a calibration curve of pyridine-2-thione (Figure S20). The results were used to calculate the yield of the conjugation reactions, which gave 87 ± 3% for the first step and 62 ± 4% for the second step (Table S1), and an overall yield of 74 ± 3% for the synthesis of PFPA-MNP-Tre from MNP-SH (Table 1).

The conjugation yield was also determined by measuring the thiol concentration on PFPA-MNP-Tre using the Ellman assay and comparing the result to that of the starting material MNP-SH. The result, calculated as the thiol% reacted, was 85 ± 8% (Table 1). The hydrodynamic diameter of PFPA-MNP-Tre increased to 10.6 ± 0.6 nm (Table 1), likely due to the large trehalose ligand and the hydration of trehalose. TEM gave a slightly smaller particle diameter of 9.8 ± 2.7 nm (Figure S21A). The surface charge of PFPA-MNP-Tre changed drastically. While MNP-SH was negatively charged with a ζ potential of −(85.9 ± 2.2) mV, the negative charge was significantly reduced after conjugation of the two ligands, giving a ζ potential of −(9.7 ± 0.5) mV for PFPA-MNP-Tre (Table 1).

PFPA-MNP-OH was synthesized following a similar procedure as PFPA-MNP-Tre (Scheme 2), except that MNP-SH was first treated with HO-SS-Py (Scheme S3B) at 0.5 equiv of the thiol concentration on MNP-SH. The conjugation yields calculated by measuring the concentration of released pyridine-2-thione byproduct were 83 ± 8 and 87 ± 5% for HO-SS-Py and PFPA-SS-Py, respectively, giving an overall yield of 85 ± 2% for the conjugation of the two ligands. The conjugation yield, determined by measuring the thiol concentration on PFPA-MNP-OH and comparing it to that of the starting material MNP-SH, was 93 ± 6% (Table 1). The hydrodynamic diameter of PFPA-MNP-OH was 8.8 ± 0.4 nm with a PDI of 1.0 ± 0.3 (Table 1), and the diameter measured by TEM was 7.0 ± 1.5 nm (Figure S22A).

Thermal gravimetric analysis (TGA) was carried out to characterize the ligand density on PFPA-MNP-Tre and PFPA-MNP-OH. In the derivative thermogravimetric (DTG) curve of PFPA-MNP-Tre, two major thermal decomposition events were observed: one having the highest rate of decomposition at ∼250 °C and the second at ∼350 °C which also overlaps with the first (red, Figure 1A). To help elucidate the nature of each event, TGA curves of the two ligands Tre-SS-Py and PFPA-SS-Py were obtained. DTG of Tre-SS-Py showed two decomposition temperatures at 225 and 250 °C (Figure S23A), which can be attributed to Py (Figure S23B) and Tre (Figure S23C), respectively. For PFPA-SS-Py, the two decompositions at 175 and 220 °C (Figure S24A) are attributed to Py (Figure S24B) and PFPA (Figure S24C), respectively. The decomposition temperatures of the free ligands are lower than those when they are tethered on the nanoparticle surface. Since the decomposition temperatures of Tre-SS-Py are higher than PFPA-SS-Py, we assign the decomposition at ∼250 °C to the loss of PFPA and at ∼350 °C to the loss of the trehalose ligand on PFPA-MNP-Tre. As the two thermal events overlap, it is difficult to calculate the density of each ligand. Nevertheless, the total ligand density of both PFPA and Tre was estimated to be 3.8 × 10^–15^ μg/nm^2^ by comparing the weight loss of PFPA-MNP-Tre to that of MNP-SH (see Figure S26A and SI for calculation). The TGA of PFPA-MNP-OH (Figure 1B) was also collected and analyzed. Similar to PFPA-MNP-Tre, the two ligands on PFPA-MNP-OH cannot be distinguished based on the TGA of two free ligands PFPA-SS-Py (Figure S24A) and HO-SS-Py (Figure S25A). Therefore, the total ligand density on PFPA-MNP-OH was estimated by comparing the weight loss of PFPA-MNP-OH to that of MNP-SH (Figure S26B and SI for calculation). The result, 3.3 × 10^–15^ μg/nm^2^, is slightly lower than that of PFPA-MNP-Tre.

**Figure 1:**
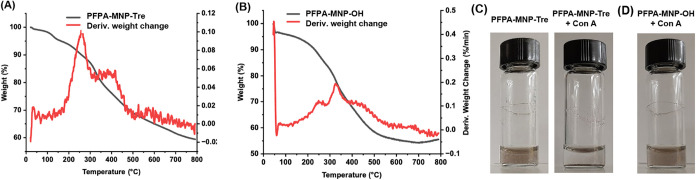
TGA (gray) and DTG (red) curves of (A) PFPA-MNP-Tre, and (B) PFPA-MNP-OH. (C) PFPA-MNP-Tre dispersed in water (0.25 mg/mL, left panel) and after incubating at 22 °C for 10 min with 0.1 mg/mL of concanavalin A (Con A) containing 1.0 mM each of MnCl_2_ and CaCl_2_ in pH 7.4 PBS buffer (right panel). (D) PFPA-MNP-OH after incubating with Con A for 1 h under the same conditions as in (C).

The presence of trehalose on PFPA-MNP-Tre was confirmed by treating with concanavalin A (Con A), a lectin that binds to carbohydrates containing glucopyranosides, with a *K*_d_ of 0.22 mM for trehalose.^57,58^ Since Con A is a tetramer, it is expected that in the presence of multivalent trehalose-presenting nanoparticles PFPA-MNP-Tre, it will form a cross-linked network leading to the formation of large agglomerates.^59–61^ Indeed, when Con A was added to the brown dispersion of PFPA-MNP-Tre (left panel, Figure 1C), precipitates formed immediately, and the supernatant was colorless (right panel, Figure 1C). For the control probe PFPA-MNP-OH, there was no change after the addition of Con A. No precipitate was observed, and the particles remained dispersed (Figure 1D).

### MAP Captured Proteins in Mycobacterial Cell Lysate

We first tested the ability of the MAP to capture proteins in the bacterial cell lysate. A nonpathogenic mycobacterium, *M. smegmatis* mc^2^155, was used. The bacteria were lysed in a lysis buffer, followed by probe sonication. After the cell debris was removed, the cell lysate containing mycobacterial proteins was incubated with MAP at 4 °C for 24 h, and the mixture was irradiated (cf. Scheme 1B). To release the captured proteins, the disulfide bonds in the linkers were cleaved by treating the samples with β-mercaptoethanol, followed by removing the nanoparticles with a magnet. The supernatant containing captured proteins were subjected to SDS-PAGE separation and analysis. Compared to the cell lysate, only a few prominent bands appeared in the samples treated with PFPA-MNP-Tre (Figure 2A). More bands appeared in the samples treated with PFPA-MNP-OH (Figure 2B).

**Figure 2:**
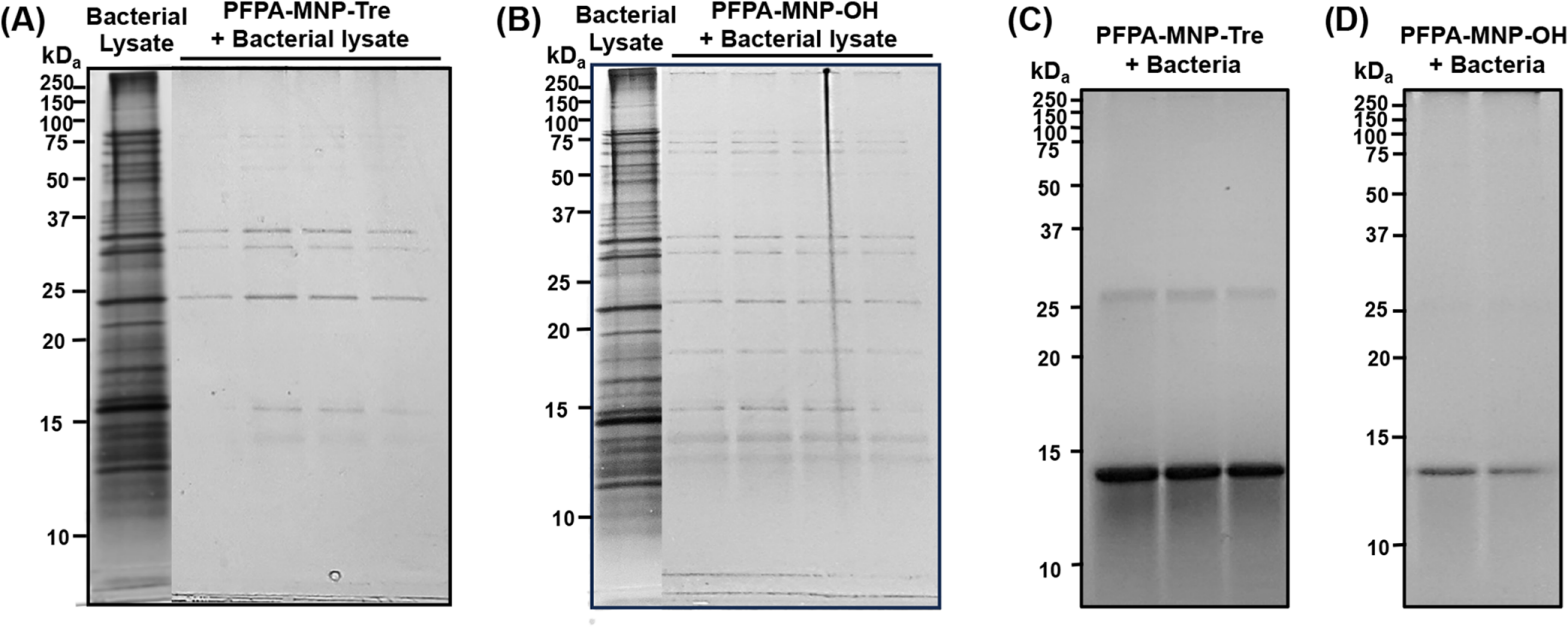
SDS-PAGE gel images of proteins captured by PFPA-MNP-Tre or PFPA-MNP-OH from (A, B) *M. smegmatis* lysate and (C, D) growth-phase *M. smegmatis*. Gels were silver-stained. The band at 14 kDa in (C) and (D) is from lysozyme in the lysis buffer.

### MAP Captured Intracellular Mycobacterial Proteins in Live Bacteria

First, a series of studies were carried out to optimize the experimental conditions, including bacterial concentration, MAP concentration, incubation time, and irradiation time, with the goal of maximizing protein capture from live bacteria, with the major protein band appearing in the region of 25–35 kDa (cf. Figure 2C) as the main focus. The optimized conditions were as follows: *M. smegmatis* concentration at 0.5 OD_600_ (∼1.5 × 10^8^ CFU/mL), MAP concentration at 0.5 mg/mL, incubation for 24 h, and irradiation at >280 nm with a medium-pressure Hg lamp (intensity = 2.5 mW/cm^2^ at 365 nm at the sample location) for 30 min (see Supporting Information and Figures S27–S30 for details). Using these conditions, the procedures outlined in Scheme 1C were followed to capture and isolate mycobacterial proteins in live mycobacteria. Briefly, *M. smegmatis* suspended in pH 7.4 PBS buffer was incubated with an equal volume of 0.5 mg/mL PFPA-MNP-Tre at 37 °C in the dark for 24 h. The sample was placed in an ice bath to dissipate heat during irradiation and was irradiated for 30 min. The bacteria were then lysed, and the nanoparticles containing the captured proteins were collected using a magnet. The disulfide bonds in the linkers were cleaved with mercaptoethanol, after which the magnetic particles were removed with a magnet, leaving behind the released bacterial proteins. The proteins were subsequently concentrated and analyzed by SDS-PAGE. Results in Figure 2C showed a band in the region between 25 and 35 kDa. No other bands could be clearly distinguished under these conditions. PFPA-MNP-OH captured a protein band in a similar molecular weight range as PFPA-MNP-Tre, however, the band intensity was lower despite using the same amounts of probe and bacteria (Figure 2D).

The protein band was then subjected to LC-MS/MS analysis (see Supporting Information for the procedure). Upon matching the peptide fragmentations with those of *M. smegmatis* mc^2^155 in the UniProt database,^62^ eight protein targets were identified (Table S3), with protein from *MSMEG_6189* being the most abundant, followed by *rpsE*, glyceraldehyde-3-phosphate dehydrogenase (GAPDH, from *gapA*), and the rest were of lower concentration. Except for *MSMEG_2079* and *MSMEG_2941*, the locations of which have not been reported, all other captured proteins are located in the cytoplasm/cytosol. Based on their molecular or biological functions, the main targets belong to proteins involved in bacterial growth and protein synthesis, such as those from *MSMEG_6189*, *prrA*, *rpsE*, and *trpA*. The other proteins from *MSMEG_2079*, *gapA*, and *MSMEG_5183* are involved in oxidative/reductive activities. GAPDH in mycobacteria was reported to be involved in the capture and acquisition of iron,^63^ an element that is present in the iron oxide nanoparticle used in MAP.

The results that the captured proteins are cytoplasmic support that PFPA-MNP-Tre was internalized in mycobacteria. On the other hand, the proteins captured are not trehalose-binding proteins. Two experiments were carried out to test the binding specificity of PFPA-MNP-Tre. In the first experiment, PFPA-MNP-Tre was treated with trehalose-binding protein Con A. The lectin peanut agglutinin (PNA), which does not bind to trehalose, was the control. At pH 7, both Con A and PNA are homotetramers having equal molecular masses of 26 ^64^ and 27 kDa,^65^ respectively. In the experiment, PFPA-MNP-Tre was incubated with Con A or PNA at 4 °C for 4 h, irradiated, and processed under the same conditions as described above to release the captured proteins. SDS-PAGE analysis showed that PFPA-MNP-Tre captured Con A but not PNA (Figure 3A). Without irradiation, PFPA-MNP-Tre could also capture Con A; however, the intensity of the band was ∼32% lower than when the sample was irradiated (Figure 3A). These results demonstrate the selective binding of PFPA-MNP-Tre to Con A over the nonbinding protein PNA.

**Figure 3:**
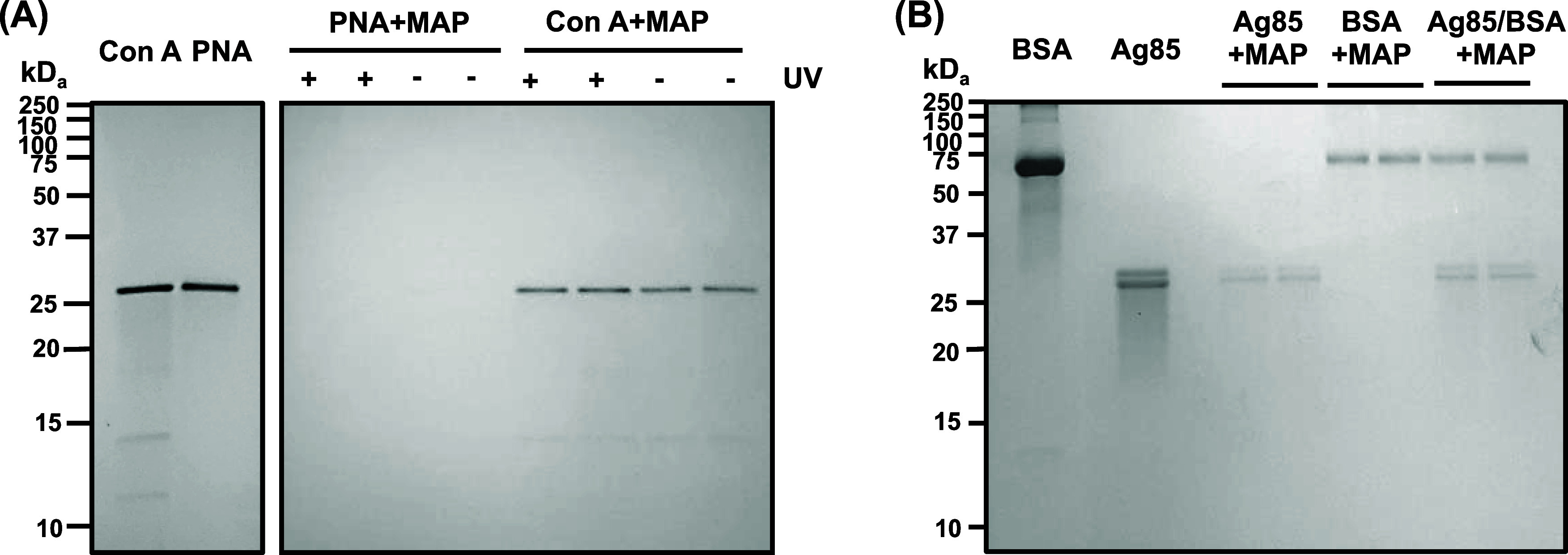
SDS-PAGE gel images of proteins captured by MAP, PFPA-MNP-Tre: (A) Con A and PNA with (+) or without (−) irradiation and (B) Ag85, BSA, or a mix of 1:1 Ag85/BSA. Samples were prepared by incubating PFPA-MNP-Tre with either the single protein or 1:1 Ag85/BSA in pH 7.4 PBS at 4 °C for 4 h. The mixture was irradiated for 30 min, and the captured proteins were isolated after the disulfide linker was cleaved with β-mercaptoethanol and the nanoparticles were removed with a magnet.

In the second experiment, PFPA-MNP-Tre was treated with the Antigen 85 (Ag85) complex, and bovine serum albumin (BSA) was the control. Ag85, found in the cell envelope of mycobacteria, catalyzes the transesterification of trehalose monomycolate to trehalose dimycolate, which is then used to build the mycobacterial cell wall.^66,67^ The Ag85 complex consists of three proteins: Ag85A (31.65 kDa), Ag85B (30.66 kDa), and Ag85C (32.02 kDa). It shows two bands in SDS-PAGE: The top band contains Ag85A and Ag85C, and the bottom band is the more abundant Ag85B (Figure 3B).^68^ BSA is a 66.46 kDa adhesive protein that tends to bind to many surfaces and materials.^69^ Results in Figure 3B show that PFPA-MNP-Tre captured both Ag85 (Ag85 + MAP) and BSA (BSA + MAP) when treated with each protein individually. We then treated PFPA-MNP-Tre with a 1:1 mix of Ag85 and BSA. Both proteins were captured in this case (Ag85/BSA + MAP, Figure 3B). The intensity of Ag85 and BSA was 1:1.9, indicating that a higher amount of BSA was captured than Ag85.

### Bacteria Were Viable after Treating with MAP

To rule out the possibility that PFPA-MNP-Tre was internalized by damaging the bacteria, the viability of the bacteria after treatment with PFPA-MNP-Tre was measured. The number of live bacteria after treating with PFPA-MNP-Tre or PFPA-MNP-OH for 24 h was assessed by plating treated bacteria on agar plates and counting the surviving colonies (see Supporting Information and Table S4). The bacterial counts of untreated *M. smegmatis* after 24 h were (2.8 ± 1.8) × 10^8^ CFU/mL. After treating with MAP for 24 h, the viable cell counts were (2.3 ± 1.8) × 10^8^ and (1.3 ± 0.1) × 10^8^ CFU/mL for PFPA-MNP-Tre and PFPA-MNP-OH, respectively. This observation that bacterial counts remained essentially unchanged indicates that there were minimal, if any, dead cells, thereby ruling out the possibility that MAP internalization occurred by damaging the bacteria.

### Impact of Trehalose on PFPA-MNP-Tre Uptake

After incubating with PFPA-MNP-Tre for 24 h, the bacteria were imaged by TEM. Results show that particles were primarily found at the poles of bacteria (Figures 4A and S31A,B). For bacteria treated with PFPA-MNP-OH, such localization was not observed, and particles were seen across the bacterial cells instead (Figures 4B and S31C,D). In mycobacteria, cell growth and division is asymmetric.^70^ The asymmetric cell division occurs predominantly at the poles due to the difference in the elongation rate.^38^ For mycobacteria, their growth and division require the synthesis of new cell wall materials at the poles.^39–43^ Trehalose is a major building block of the mycobacterial cell wall, involved in the synthesis of the glycolipid trehalose mycolates. Studies using trehalose-based molecular probes have provided strong evidence of the involvement of trehalose in mycobacterial growth and division occurring at the poles. For example, Bertozzi and co-workers treated *M. smegmatis* with azide-tagged trehalose, and after reacting with alkyne-derivatized Alexa Fluor 488, fluorescence was observed mainly at the poles where new cell wall biosynthesis takes place.^71^ This polar labeling in *M. smegmatis* has been consistently reported, for example, in the study of mycobacterial cell wall using trehalose glycolipids,^72^ in metabolic tagging of trehalose mycolates,^73^ and in the study using a trehalose-based fluorescent probe to directly visualize the accumulation of the probe at the bacterial poles and septa.^74^ We thus postulate that the accumulation of PFPA-MNP-Tre at the mycobacterial poles might be the consequence of the high requirement of nutrients, such as trehalose, at the poles. In an initial test of this hypothesis, *M. smegmatis* was incubated with PFPA-MNP-Tre in the presence of varying concentrations of free trehalose. As shown in Figure 4C, the intensity of the protein band decreased with an increasing concentration of free trehalose. This observation suggests that internalization of PFPA-MNP-Tre may involve the trehalose uptake pathway, however, additional experiments are required to confirm the mechanism.

**Figure 4:**
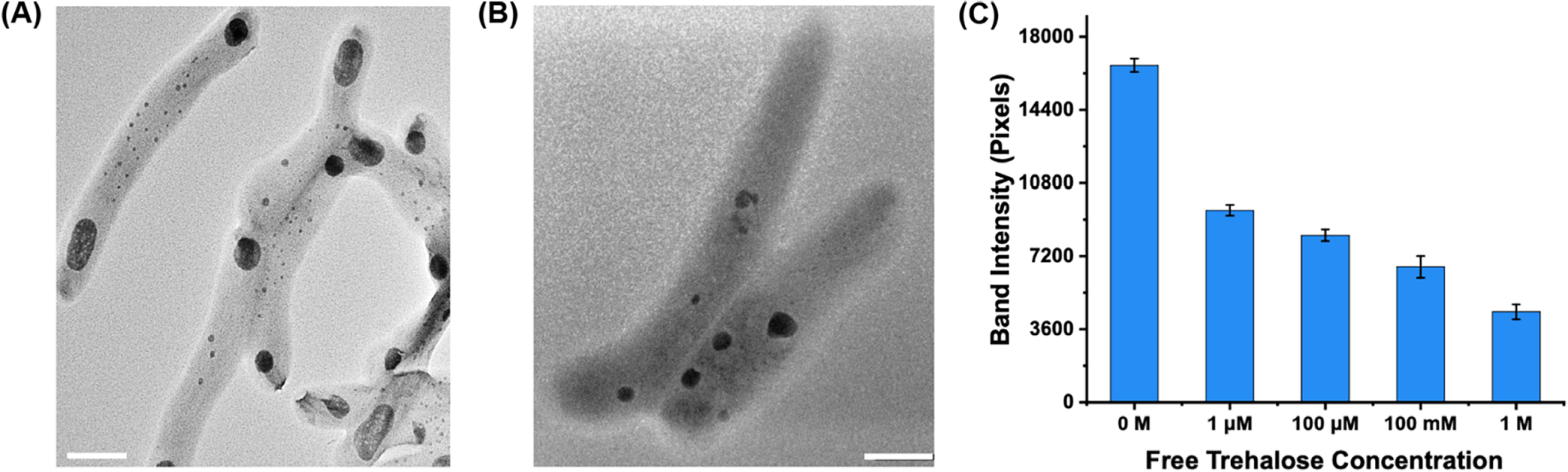
TEM images of *M. smegmatis* after treatment with (A) PFPA-MNP-Tre or (B) PFPA-MNP-OH for 24 h. Scale bars: 500 nm. Additional images can be found in Figure S31. (C) Intensity of the captured 25–35 kDa protein band vs concentration of added free trehalose. The band intensity was measured using ImageJ. Data were the average from two independent trials. The experimental procedure can be found in the Supporting Information. The gel images are shown in Figure S32.

## Discussion

TEM, ICP-MS/ICP-AES/AAS, SPR, fluorescence imaging, and flow cytometry are among the most commonly used techniques to study the internalization of nanoparticles in bacteria; however, these techniques provide indirect evidence. In this work, we developed a new approach to characterize nanoparticle internalization by conjugating a photoaffinity labeling agent on the nanoparticle to enable the capture of nanoparticle-binding proteins in bacteria. Subsequent proteomic analysis of captured proteins provides direct biochemical evidence for nanoparticle internalization.

The model MAP consists of magnetic nanoparticles functionalized with PFPA and trehalose or ethanol (Scheme 1A). The iron oxide magnetic nanoparticle was chosen for the ease of synthesis, separation, and purification. For both ligands, a disulfide linker was introduced, so that it can be subsequently cleaved to release captured proteins. MAP was synthesized by functionalizing the iron oxide nanoparticles with a thiol to give MNP-SH, followed by disulfide exchange with pyridine disulfide-derivatized ligands Tre-SS-Py or HO-SS-Py and then PFPA-SS-Py (Scheme 2). The ligand conjugation reaction can be conveniently monitored by measuring the released pyridine-2-thione, from which the conjugation yield was calculated (Table 1). The yield was also determined by measuring the thiol concentration on MNP-SH before and after ligand conjugation (Table 1).

Following the general protocol in Scheme 1C, we successfully used MAP to capture and isolate bacterial proteins from live bacteria. Proteomic analysis revealed that identified proteins captured by PFPA-MNP-Tre were cytoplasmic and were related to bacterial growth or the redox response (Table S3). A major protein band in the region of 20–35 kDa was captured from live bacteria (Figure 2C), whereas multiple protein bands were captured from the bacterial lysate (Figure 2A). In bacterial lysates, both membrane-associated and cytoplasmic proteins are present, whereas only cytoplasmic proteins are available for the internalized nanoparticles. The ability of PFPA-MNP-Tre to capture cytoplasmic proteins demonstrates the unique advantage of the MAP strategy: spatiotemporal control and the ability to capture the binding proteins on demand by photoactivation.

Nonspecific protein binding was seen in the case of BSA, where it was captured at a higher amount in the presence of an equal concentration of Ag85 (Figure 3B). The observation that PFPA-MNP-Tre bound Con A without irradiation indicates that PFPA-MNP-Tre interacted with Con A through nonspecific adsorption. The observation that PFPA-MNP-Tre bound both Ag85 and BSA indicates nonspecific adsorption of PFPA-MNP-Tre in its current design.

Several results demonstrate the significant effect of surface ligands on the uptake of nanoparticles in bacteria: (1) PFPA-MNP-Tre (Figure 2A) captured different sets and/or amount of proteins than PFPA-MNP-OH (Figure 2B); (2) PFPA-MNP-Tre captured higher mount of proteins (Figure 2C) than PFPA-MNP-OH under identical experimental conditions (Figure 2D); (3) PFPA-MNP-Tre accumulated at the poles (Figure 4A) whereas PFPA-MNP-OH were seen across the bacterial cells (Figure 4B). These results further validate the strategy of using ligands to modulate the interactions and uptake of nanoparticles by bacteria. The observations that PFPA-MNP-Tre bound Con A without irradiation, albeit in a lower amount (Figure 3A), and PFPA-MNP-Tre bound to both Ag85 and BSA (Figure 3B) indicate that the current probe is prone to nonspecific protein adsorption. Future work will focus on improving probe design, for example, by optimizing linker structure and ligand density, to minimize nonspecific adsorption.

## Conclusions

In conclusion, we have developed and synthesized magnetic affinity probes to capture intracellular proteins in live bacteria. The MAP design includes magnetic nanoparticles and a cleavable linker to facilitate protein isolation and purification. Using PFPA and trehalose as the model ligands, we synthesized PFPA-MNP-Tre and subsequently used it to capture and isolate mycobacterial proteins. Proteomic analysis showed that at 24 h incubation, the captured proteins are cytoplasmic, thus providing direct biochemical evidence for the internalization of nanoparticles. Future work will focus on improving the probe design, for example, by optimizing linker structure and PFPA/ligand ratio, to minimize nonspecific adsorption. As the MAP platform is modular, where both the photoaffinity label and the ligand can be tailored to the system under investigation, it may find broad applications in studying mechanisms and processes involving nanoparticle–cell interactions.

## Experimental Section

### Materials and Instrumentation

Reagents and solvents purchased from Fisher Scientific, Sigma-Aldrich, or Alfa Aesar were used as received without further purification unless otherwise noted. Reactions were monitored by thin-layer chromatography (TLC) using TLC plates precoated with silica gel 60 F254 (SiliCycle, Inc.) and visualized with a hand-held UV lamp directly or after staining with 5% H_2_SO_4_ solution in ethanol. Compounds were dried in a VWR symphony vacuum oven or a Labconco FreeZone 2.5 freeze-dryer. The pH 7.4 phosphate-buffered saline (PBS) was prepared by dissolving a PBS tablet in deionized water (1 L) followed by autoclaving (Tuttnauer EZ10, Hauppauge, NY). The Sauton medium was prepared by mixing KH_2_PO_4_ (0.5 g), MgSO_4_ (0.5 g), l-asparagine (4.0 g), glycerol (60 mL), ferric ammonium citrate (0.05 g), citric acid (2.0 g), ZnSO_4_ (0.1 mL, 1% w/v) in 900 mL of Milli-Q water supplemented with Tween-80 (0.05%), adjusting to pH 7 with 10 M KOH followed by autoclaving. Laemmli buffer was prepared by mixing 65.8 mM pH 6.8 Tris–HCl, 2.1% sodium dodecyl sulfate (SDS), 26.3% (w/v) glycerol, and 0.01% bromophenol blue.

^1^H, ^13^C and ^19^F NMR spectra were recorded on a BrukerAvance Spectrospin DRX500 spectrometer or a JEOL ECZ 400 MHz NMR. ^19^F NMR spectra were recorded by using CF_3_COOH as the external standard. Infrared spectra were recorded on a Thermo Electron NICOLET 6700FT-IR spectrometer using a diamond ATR accessory. Mass spectra of compounds were obtained on a Waters Xevo G2-XS Quadrupole Time-of-Flight Mass Spectrometer. LC-MS/MS of protein samples was performed on a Velos Orbitrap Pro ion-trap mass spectrometer (Thermo Fisher Scientific, Waltham, MA) at the Taplin Mass Spectrometry facility at Harvard University. TGA was carried out on a TA Instruments Q series Thermogravimetric analyzer. Samples were first heated to 100 °C under N_2_ for 10 min and, after cooling down to room temperature, were then heated to 800 °C at a rate of 20 °C/min.

Synthesis and characterization data of MNP, MNP-SH, Tre-SS-Py, PFPA-SS-Py, and HO-SS-Py can be found in the Supporting Information.

### Synthesis of PFPA-MNP-Tre and PFPA-MNP-OH

PFPA-MNP-Tre was synthesized in two steps by treating MNP-SH with Tre-SS-Py followed by PFPA-SS-Py (Scheme 2). The following is a typical procedure. A solution of Tre-SS-Py (1.67 mg, 3.57 × 10^–3^ mmol) in 0.5 mL of methanol was added to MNP-SH (60 mg, [SH] = 7.14 × 10^–3^ mmol) dispersed in 4.5 mL of methanol. The mixture was stirred at room temperature at 1000 rpm. Every 6 h, a volume of 500 μL of the reaction mixture was withdrawn and centrifuged to sediment the particles. The absorbance of the supernatant was measured at 343 nm until the absorbance was unchanged, which took about 24 h. Afterward, particles were purified by repeated washing with methanol followed by centrifugation 5 times to remove any unbound ligands. The resulting particles were dispersed in 4.5 mL of methanol, and a solution of PFPA-SS-Py (1.45 mg, 3.57 × 10^–3^ mmol) in 0.5 mL of methanol was added. The mixture was stirred at 1000 rpm at room temperature. A volume of 500 μL of the reaction mixture was withdrawn every 6 h and centrifuged, and the absorbance of the supernatant was measured at 343 nm. The absorbance increased initially and then remained unchanged after 24 h. Particles were purified by repeated washing and centrifugation 5 times with methanol to remove any unbound ligands. The purified particles were finally dried to give PFPA-MNP-Tre (56 mg).

PFPA-MNP-OH was synthesized following the same procedure as that for PFPA-MNP-Tre. MNP-SH (50 mg, [SH] = 6.0 × 10^–3^ mmol) dispersed in 4.5 mL of methanol was mixed with a solution of HO-SS-Py (0.56 mg, 3.0 × 10^–3^ mmol) in 0.5 mL of methanol and was stirred at 1000 rpm at room temperature for 24 h. After purification by repeated washing with methanol followed by centrifugation 5 times, the particles were dispersed in 4.5 mL of methanol, and a solution of PFPA-SS-Py (1.20 mg, 3.0 × 10^–3^ mmol) in 0.5 mL of methanol was added. The mixture was stirred at 1000 rpm at room temperature for 24 h, purified by repeated washing and centrifugation 5 times with methanol, and dried to give PFPA-MNP-OH (48 mg).

### Capture Proteins from *M. smegmatis* Lysate with MAP

Bacterial lysate was prepared as follows. *M. smegmatis* mc^2^155 was cultured in 10 mL of Sauton medium until it reached mid-logarithmic phase to an optical density (OD_600_) of 0.5 (Figure S33), which corresponds to bacterial counts of 1.5 × 10^8^ CFU/mL.^75^ The lysis buffer (1 mL), prepared by mixing 0.3 mg/mL lysozyme and 1 mM phenylmethylsulfonyl fluoride (PMSF) protease inhibitor in pH 7.4 PBS buffer, was added and incubated at 37 °C for 0.5 h. The mixture was then subjected to probe sonication for 2 min (10 s ON and 30 s OFF intervals at 20% amplitude) in an ice bath. SDS was added to the lysate at a final concentration of 2% (w/v), incubated at 60 °C for 15 min, and then in ice for 1 h. The mixture was centrifuged at 5000 rpm at 4 °C for 15 min. After the bacterial debris was removed, the supernatant was transferred to a 15 mL tube. Cold acetone (−20 °C) was added at four times the volume of the supernatant, vortexed, and then stored at −20 °C overnight for proteins to precipitate. The mixture was centrifuged at 9000 rpm and 4 °C for 10 min. The cold acetone wash and centrifugation steps were repeated twice more, and the supernatant was decanted. Acetone was evaporated under ambient conditions without letting the protein pellet dry completely. The pellet was redispersed in 5 mL of cold PBS buffer, and a volume of 250 μL was added to each well of a 24-well plate, followed by 250 μL of 0.5 mg/mL MAP in PBS. The mixture was incubated at 4 °C for 24 h while shaking at 200 rpm in the dark. Samples were centrifuged at 10,000 rpm for 5 min and washed twice with PBS. After transferring into a glass vial, the sample was covered with a 280 nm long-pass optical filter and was irradiated in an ice bath for 30 min using a 450-W medium-pressure Hg lamp (intensity at 365 nm: 2.5 mW/cm^2^ at the sample location). A magnet was applied to precipitate the particles, which were then washed three times with PBS. The final particles were dispersed in 10 μL of PBS.

### Capture *M. smegmatis* Proteins in Live Mycobacteria

A single colony of *M. smegmatis* mc^2^155 from a streaked LB agar plate was inoculated into 3 mL liquid Sauton medium in a culture tube and incubated at 37 °C while shaking until OD_600_ reached 0.5, which corresponds to 1.5 × 10^8^ CFU/mL.^75^ The bacterial suspension was centrifuged at 5000 rpm for 10 min, and the resulting pellet was redispersed in PBS to an OD_600_ of 0.5. An aliquot of 250 μL was then incubated with MAP (250 μL, 0.5 mg/mL) in a 24-well plate at 37 °C for 24 h in the dark while shaking at 200 rpm. The mixture was centrifuged at 10,000 rpm for 5 min, and the precipitate was washed twice with PBS. The pellet was redispersed in 500 μL PBS, transferred to a glass vial, which was then cooled in an ice bath, covered with a 280 nm long-pass optical filter, and irradiated for 30 min. The particles were separated with a magnet, washed with PBS three times and transferred to a 15-mL polypropylene tube. The lysis buffer (1 mL) was added, incubated at 37 °C for 30 min, and probe sonicated for 2 min in an ice bath. Finally, SDS was added to a final concentration of 2% (w/v). The homogenized cell extracts were incubated at 60 °C for 15 min and then in ice for 1 h. The particles were precipitated by a neodymium magnet. The supernatant containing cell debris was decanted, and the precipitate was washed three times with PBS. The final particles were dispersed in 10 μL of PBS buffer.

### Isolation of Captured Proteins from Nanoparticles

Captured proteins were released from the nanoparticles by cleaving the disulfide bond in the surface ligands, following the procedure below. To 3 μL of purified particles dispersed in PBS, 3 μL of 2× Laemmli buffer containing 2.5% β-mercaptoethanol was added. The mixture was heated to 95 °C for 5 min to ensure complete reduction of the disulfide bonds. While the nanoparticles were held with a magnet, the supernatant containing the released proteins was transferred to a clean tube. This treatment was repeated two additional times to maximize the protein recovery. The three supernatants were combined (∼18 μL total, 6 μL per extraction), loaded onto a 14% polyacrylamide gel, and electrophoresed at 150 V for 1.5 h. Protein bands were visualized by silver staining of the gel.

### Capture Single and Mixed Proteins with MAP

Individual protein solutions (Con A, PNA, Ag85, and BSA) were prepared at a concentration of 9.2 μM in pH 7.4 PBS buffer. For the mixed protein solution (1:1 Ag85/BSA), both Ag85 and BSA were prepared at 4.6 μM each. For the protein capture experiment, 100 μL each of the protein solution and the MAP suspension (1 mg/mL in pH 7.4 PBS) were mixed in a 96-well plate and incubated at 4 °C in the dark while shaking at 200 rpm for 4 h. The nanoparticles were then separated using a magnet, washed three times with PBS, and redispersed in 200 μL of PBS. After irradiation in the presence of a 280 nm long-pass optical filter in an ice bath for 30 min, the nanoparticles were washed three times with PBS with the aid of the magnet and were finally dispersed in 3 μL of PBS. Control samples without irradiation were prepared under identical conditions, except that the irradiation step was omitted. Protein release, isolation, and subsequent SDS-PAGE analysis were carried out following the same protocol described above under “Isolation of Captured Proteins from Nanoparticles”.

## Supplementary Material



## Notes

Synthesis and characterization data of MNPs, MNP-SH, Tre-SS-Py, PFPA-SS-Py, and HO-SS-Py, quantification of thiol concentration using Ellman assay, calibration of pyridine-2-thione, thiol concentration and ζ potential of nanoparticle samples, additional characterization data of PFPA-MNP-Tre and PFPA-MNP-OH, additional TGA data and calculations of ligand density, optimization of conditions for capturing *M. smegmatis* proteins, protocols for analysis and identification of proteins by LC-MS/MS, determination of bacteria viability, experimental procedure of TEM imaging and additional TEM images, competition with free trehalose (PDF)
