# Supplementary material for: Trehalose-Functionalized Magnetic Affinity Probe Provides Biochemical Evidence of Nanoparticle Internalization in Mycobacteria
Source: ACS Infect Dis. 2025 Sep 26;11(10):2847–58. doi: 10.1021/acsinfecdis.5c00506 (PMC12519468; doi:10.1021/acsinfecdis.5c00506)
Supplement: Supplementary file 1 [file id5c00506_si_001.pdf]

Supporting Information

Trehalose-Functionalized Magnetic Affinity Probe Provides Biochemical Evidence of Nanoparticle Internalization in Mycobacteria

Harini A. Perera,[1] *N.G. Hasitha Raviranga,* [1] *Olof Ramström,* [1,2] *Mingdi Yan*[1]*

[1]Department of Chemistry, University of Massachusetts Lowell, One University Ave., Lowell, MA 01854, USA

[2]Department of Chemistry and Biomedical Sciences, Linnaeus University, SE-39182 Kalmar, Sweden

Table of Contents

1. Synthesis of iron oxide magnetic nanoparticles, MNPs S2
2. Synthesis of thiol-functionalized iron oxide nanoparticles, MNP-SH S2
3. Synthesis of PFPA-SS-Py S3
4. Synthesis of Tre-SS-Py S9
5. Quantification of thiol concentration on nanoparticles using Ellman assay S15
6. Calibration of pyridine-2-thione S17
7. Ligand conjugation yields S17
8. TEM image and FT-IR spectrum of PFPA-MNP-Tre S18
9. TEM image and FT-IR spectrum of PFPA-MNP-OH S19
10. TGA data S19
11. Calculation of ligand density on PFPA-MNP-Tre and PFPA-MNP-Tre S21
12. Optimization of conditions for capturing *M. smegmatis* proteins with PFPA-MNP-Tre in live bacteria. S22
13. Identification of isolated proteins by LC-MS/MS S24
14. Determination of bacteria viability S26
15. TEM imaging S26
16. Competition with free trehalose S27
17. Growth curve of *M. smegmatis* mc^{2}155 ..... S28
18. References S28

1. Synthesis of iron oxide magnetic nanoparticles, MNPs

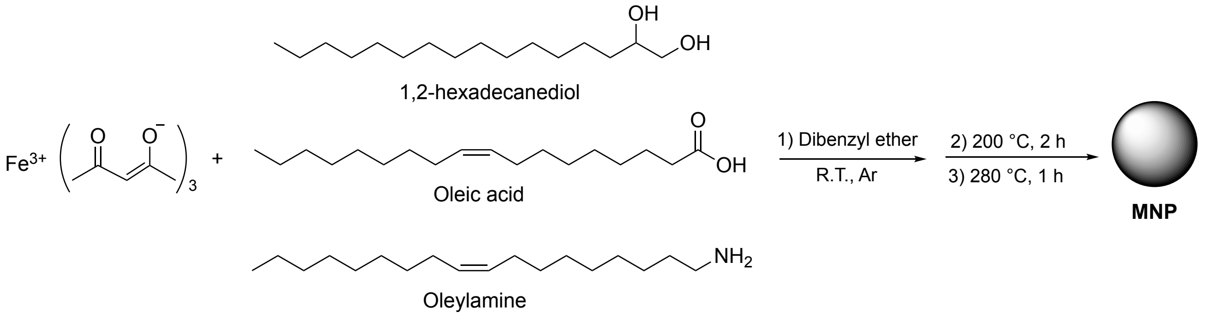

Scheme S1. Synthesis of MNPs.

A previously reported procedure was adapted.[1] Iron(III) acetylacetonate (0.71 g, 2.0 mmol), 1,2-hexadecanediol (2.6 g, 10 mmol), oleic acid (2.2 mL, 6.0 mmol) and oleylamine (2.8 mL, 6.0 mmol) were added to dibenzyl ether (30 mL), and the solution was stirred at room temperature under Ar protection. The mixture was then heated to 200 °C for 2 h and maintained at 280 °C for another hour. Ethanol (50 mL, 200-proof) was added after cooling down to room temperature and the mixture was centrifuged at 7000 rpm for 10 min. The black precipitate was re-dispersed in hexanes (30 mL) containing oleic acid (0.05 mL) and oleylamine (0.05 mL), and was centrifuged at 6000 rpm for 10 min. The precipitate was discarded, and the supernatant was collected, and ethanol was added. After centrifugation, the precipitate (150 mg) was re-dispersed in hexanes at 100 mg/mL concentration until further use.

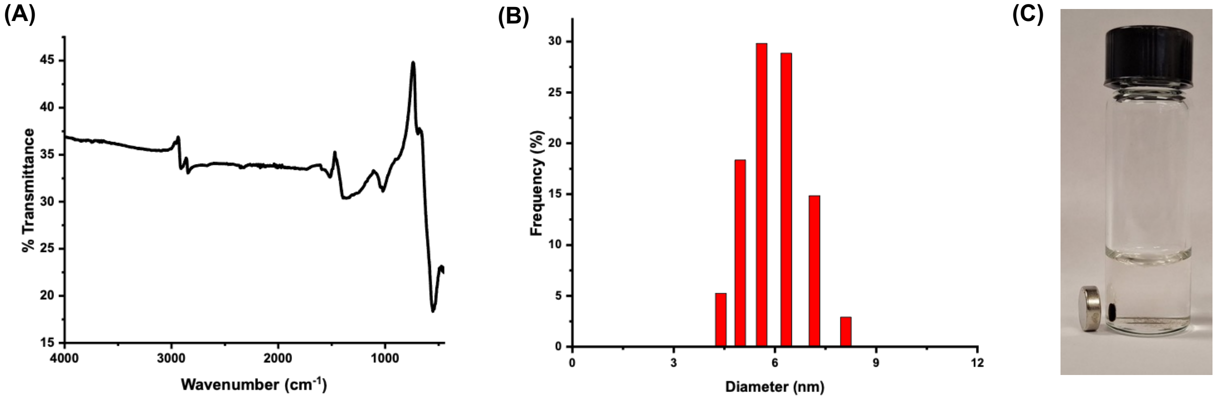

Figure S1. (A) FT-IR spectrum of MNPs. The strong broad band at 570 cm^{-1} was from the Fe-O stretching.[2] (B) DLS histogram of MNPs. (C) Precipitation of MNPs using a magnet.

2. Synthesis of thiol-functionalized iron oxide nanoparticles, MNP-SH

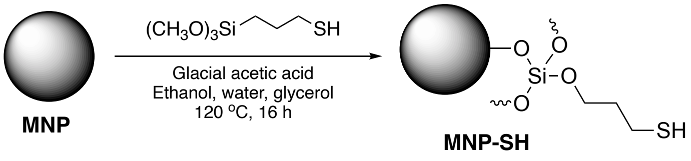

Scheme S2. Synthesis of **MNP-SH**.

A previously reported protocol was adapted.[3] MNPs (80 mg) suspended in 200-proof ethanol (4 mL) were mixed with 100 µL of water and 200 µL glacial acetic acid. Then, (3-mercaptopropyl)trimethoxysilane (MPTMS, 400 µL, 2.15 mmol) was added. The suspension was sonicated for 30 min and subsequently

transferred into a round bottom flask containing 12 mL of glycerol. The reaction mixture was heated to 120 °C in N_2 and was stirred under reflux for 16 h. The particles were precipitated by applying a magnet to the reaction flask. The precipitate was then washed 5 times with water and 5 times with ethanol. After drying under reduced pressure, the product MNP-SH was obtained as a black powder (76 mg).

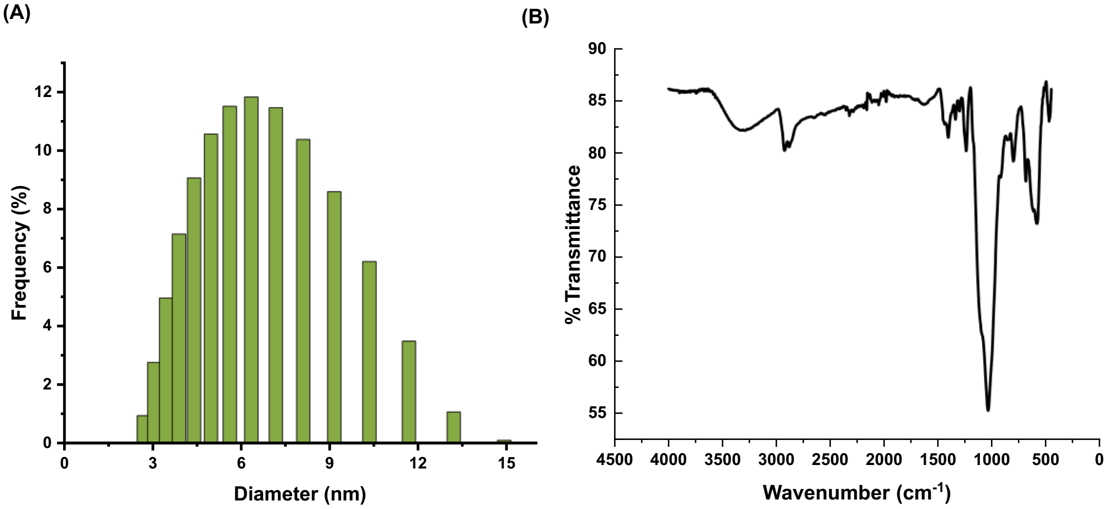

Figure S2. (A) DLS histogram of MNP-SH. (B) FT-IR spectrum of MNP-SH.

3. Synthesis of PFPA-SS-Py

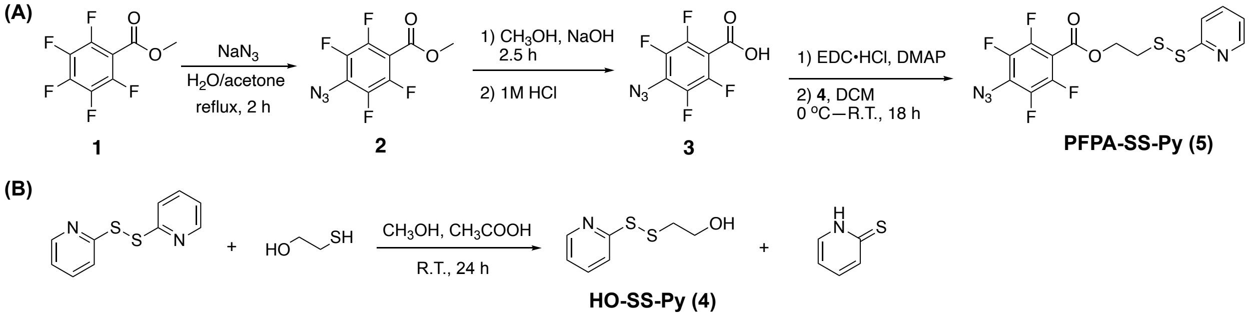

Scheme S3. Synthesis of (A) PFPA-SS-Py, (B) HO-SS-Py.

Synthesis of methyl 4-azido-2,3,5,6-tetrafluorobenzoate (2)[4]

Methyl pentafluorobenzoate (**1**, 2.00 mL, 13.6 mmol) was dissolved in a 1:2 (v/v) mixture of water and acetone (30 mL). After adding sodium azide (1.15 g, 17.6 mmol) to the flask, the solution was refluxed at 90 °C for 2 h. The mixture was subsequently cooled to room temperature, and water (60 mL) was added. After extracting with diethyl ether (60 mL × 3), the combined extracts were dried over anhydrous Na_2SO_4. The solvent was then evaporated under reduced pressure to yield product **2** as white crystals (3.4 g, 96%). ^1H NMR ($CDCl_3$): δ 3.97 (s, 3 H).

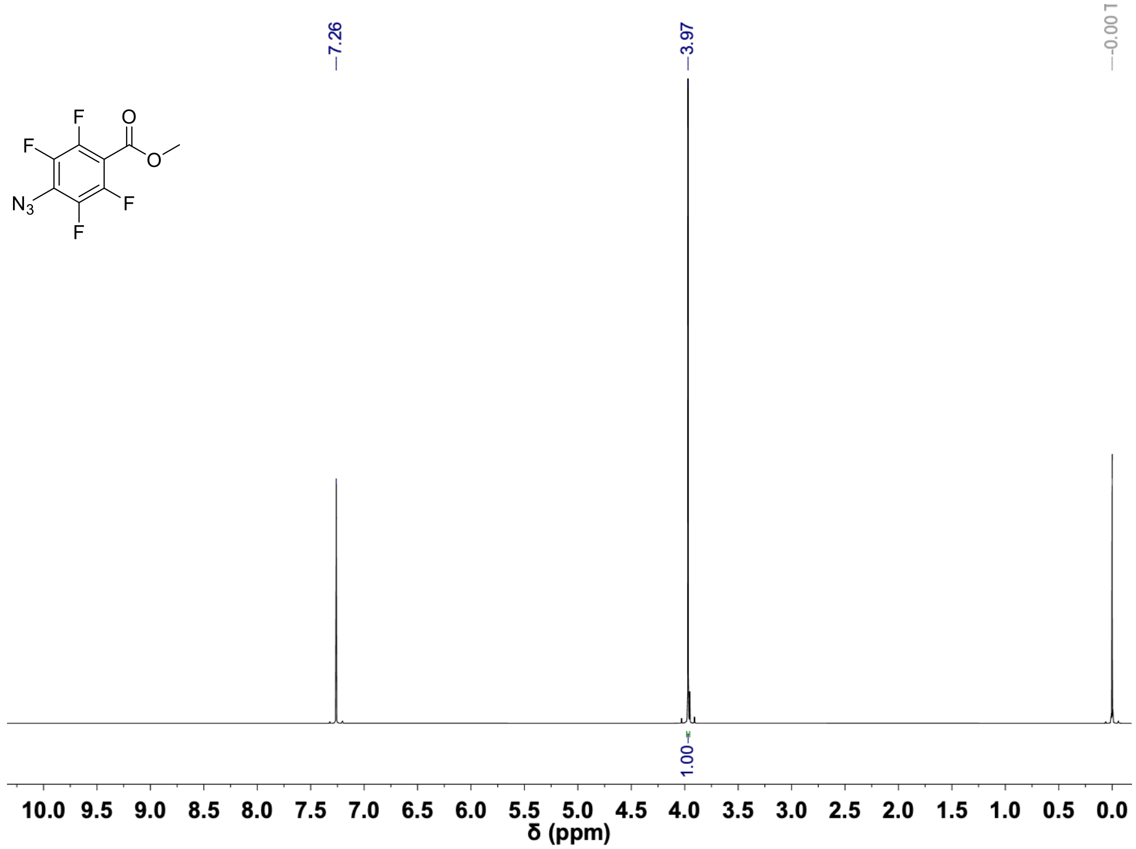

Figure S3. ^{1}H NMR spectrum of compound **2** in $CDCl_3$.

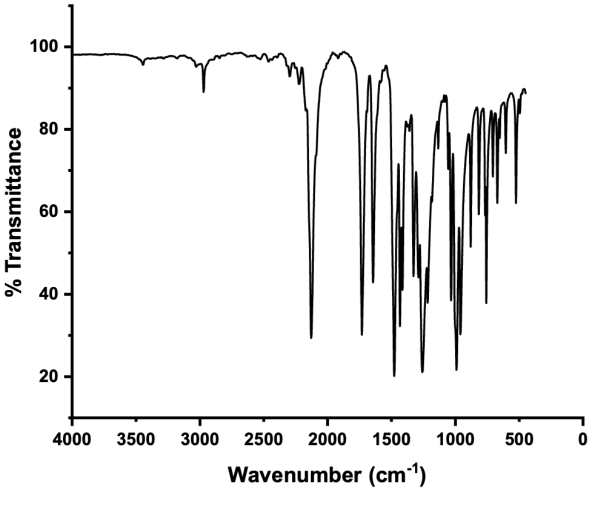

Figure S4. FT-IR (ATR) spectrum of compound **2**.

Synthesis of 4-azido-2,3,5,6-tetrafluorobenzoic acid (3)[4]

Compound **2** (3.86 g, 15.5 mmol) was dissolved in methanol (14.6 mL), and the solution was added to an aqueous sodium hydroxide solution (20%, 1.5 mL) and water (3.1 mL). The solution was then stirred at room temperature for 2.5 h, acidified with 1 M aqueous HCl to pH 1 and extracted with dichloromethane (60 mL × 3). After drying over anhydrous Na_2SO_4, the solvent was evaporated under reduced pressure, yielding product **3** as light-yellow crystals (3.4 g, 94%). ^{13}C NMR ($CDCl_3$): δ 163.73, 147.07, 144.99, 141.44, 139.46, 124.69, 106.01.

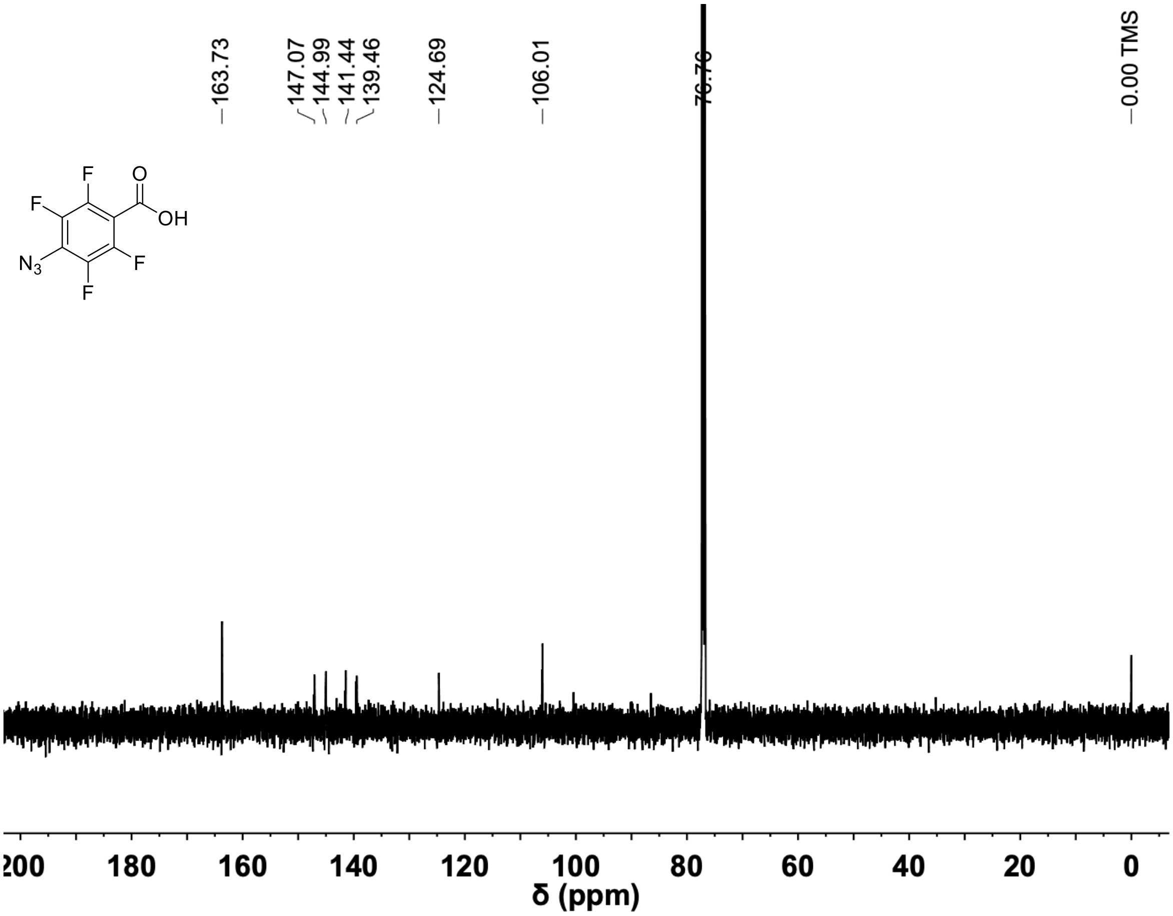

Figure S5. ^{13}C NMR spectrum of compound **3** in $CDCl_3$.

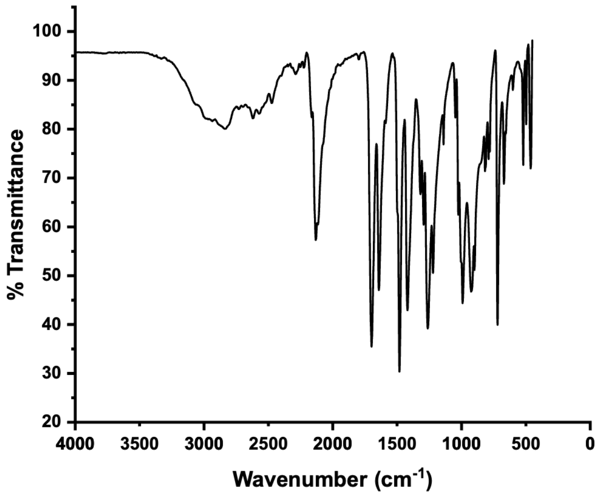

Figure S6. FT-IR (ATR) spectrum of compound **3**.

Synthesis of 2-(pyridine-2-yldisulfanyl)ethanol (4)[5]

2,2'-Dithiopyridine (2.50 g, 11.3 mmol) was dissolved in methanol (25 mL), and acetic acid (1.5 mL) was added. A solution of 2-mercaptoethanol (0.40 mL, 5.0 mmol) dissolved in methanol (10 mL) was added dropwise, and the solution was stirred at room temperature for 24 h. The solvent was removed under reduced pressure, and the crude product was purified by column chromatography using 3:2 v/v hexanes:ethyl acetate as the eluent to give product **4** as a white solid (2.0 g, 93%). ^{1}H NMR ($CDCl_3$): δ 8.52 (m, 1H), 7.58 (m, 1H), 7.41 (m, 1H), 7.15 (m, 1H), 3.81 (m, 2H), 2.96 (m, 2H).

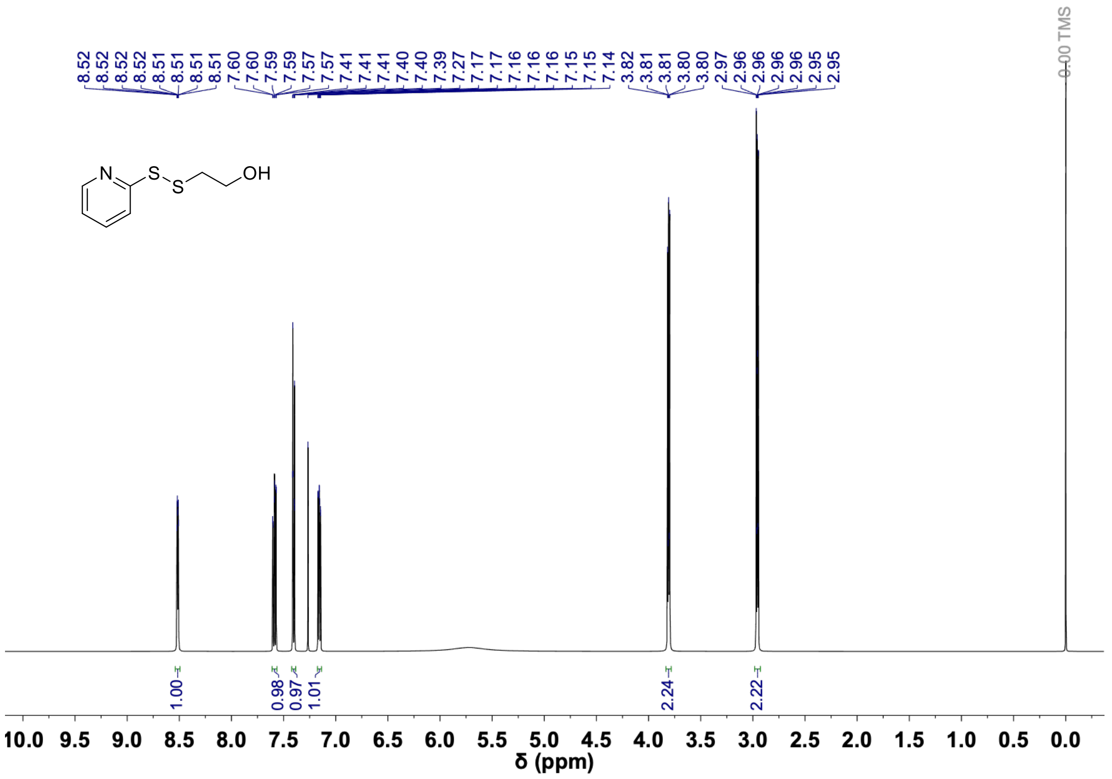

Figure S7. ^{1}H NMR spectrum of compound **4** in $CDCl_3$.

Synthesis of PFPA-SS-Py

Synthesis was performed by following a previously reported procedure.[6] Compound **4** (187 mg, 1.0 mmol), 4-(dimethylamino)pyridine (DMAP, 12 mg, 0.098 mmol) and 1-ethyl-3-(3-dimethylaminopropyl)carbodiimide (EDC·HCl, 211 mg, 1.1 mmol) were added to a solution of compound **3** in dichloromethane (1.5 mL) at 0 °C. The solution was allowed to warm to room temperature and was stirred for 18 h. Purification by column chromatography with 1:1 v/v hexanes:ethyl acetate afforded product **PFPA-SS-Py** as a light yellow solid (184 mg, 46%). ^1H NMR ($CDCl_3$): δ 8.48 (d, 1H), 7.69 – 7.62 (m, 2H), 7.11 (t, 1H), 4.63 (t, 2H), 3.16 (t, 2H). ^{19}F NMR ($CDCl_3$): δ -150.63, -137.99.

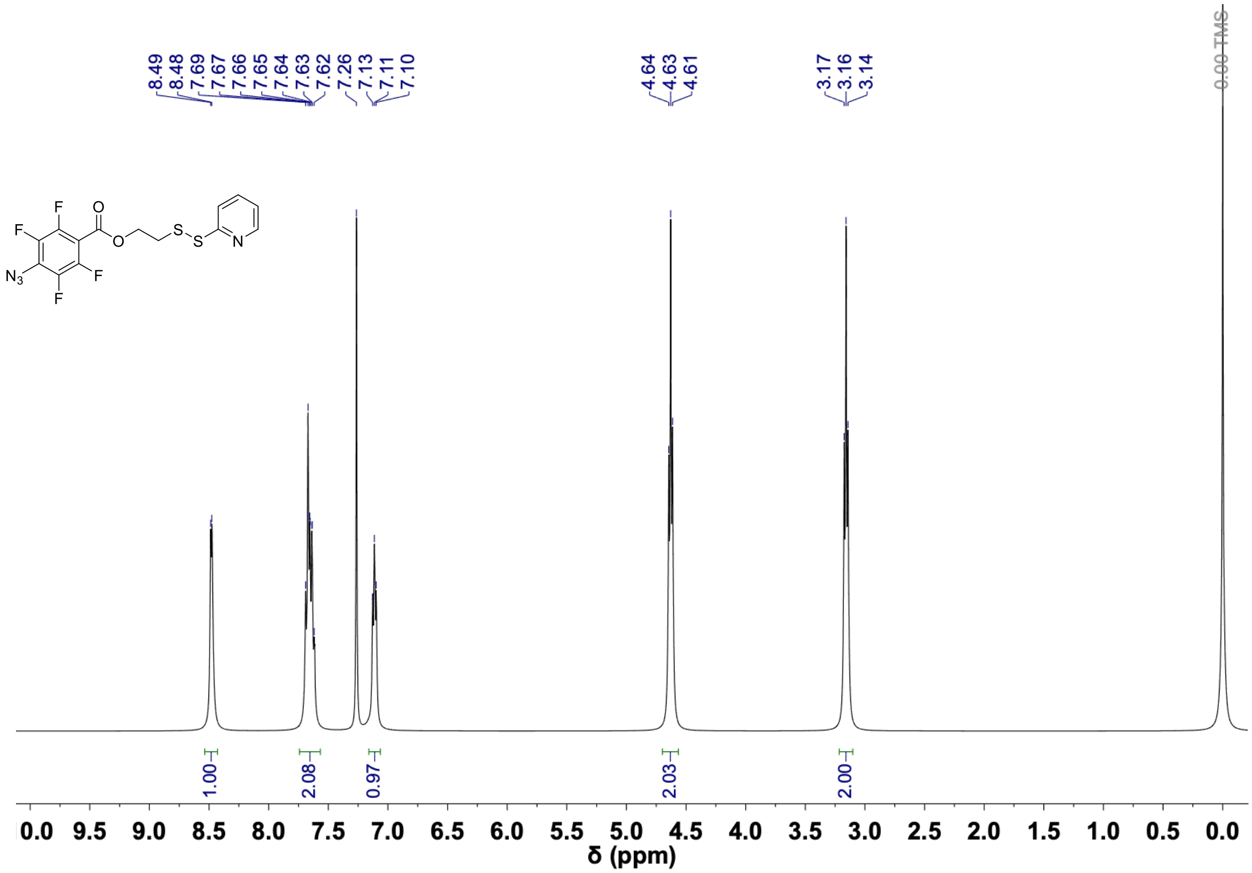

Figure S8. ^1H NMR spectrum of **PFPA-SS-Py** in $CDCl_3$.

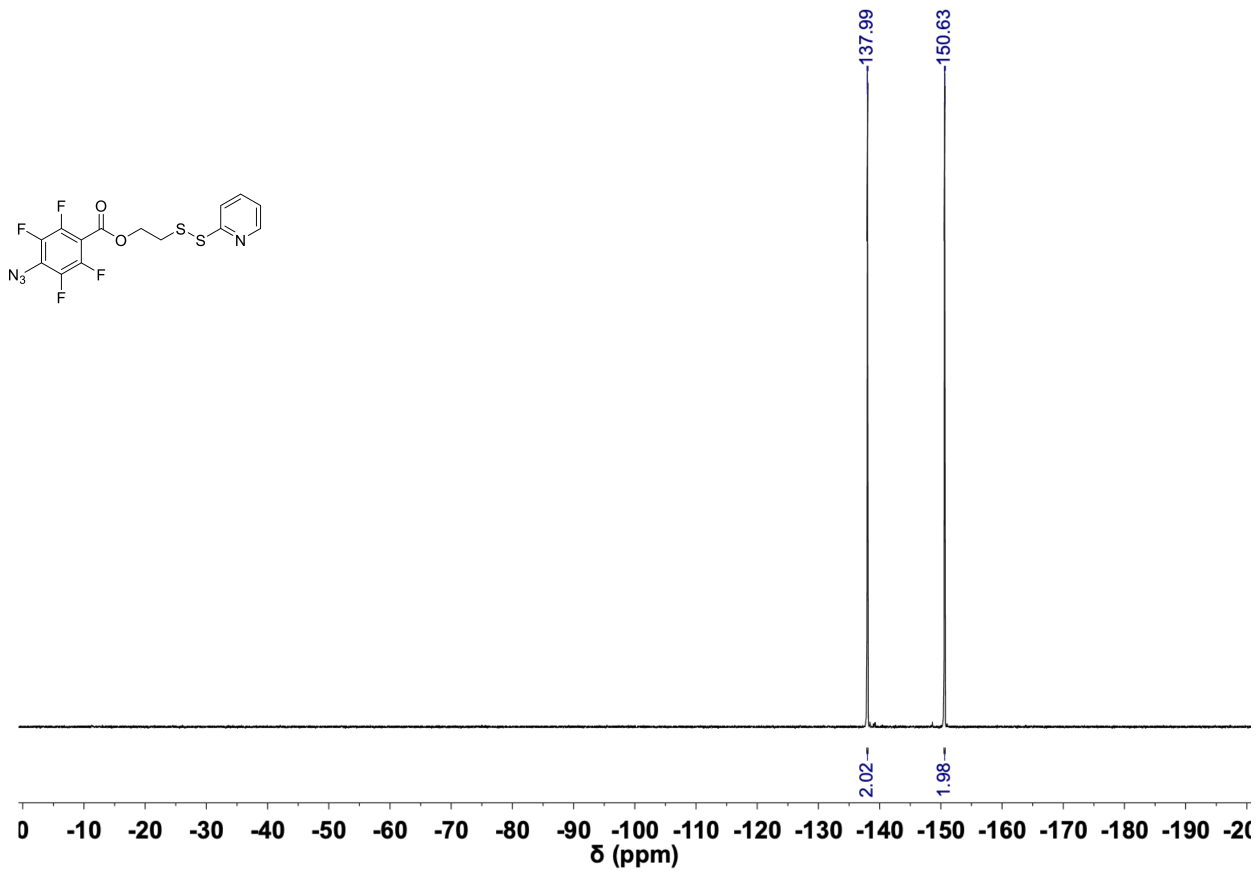

Figure S9. ^{19}F NMR spectrum of **PFPA-SS-Py** in $CDCl_3$.

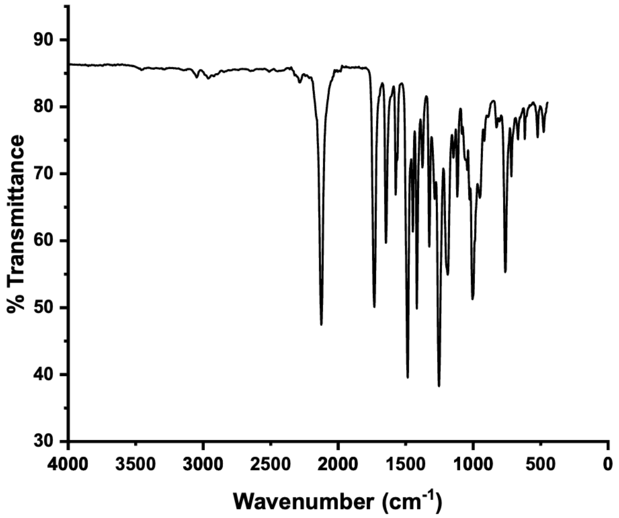

Figure S10. FT-IR (ATR) spectrum of **PFPA-SS-Py**.

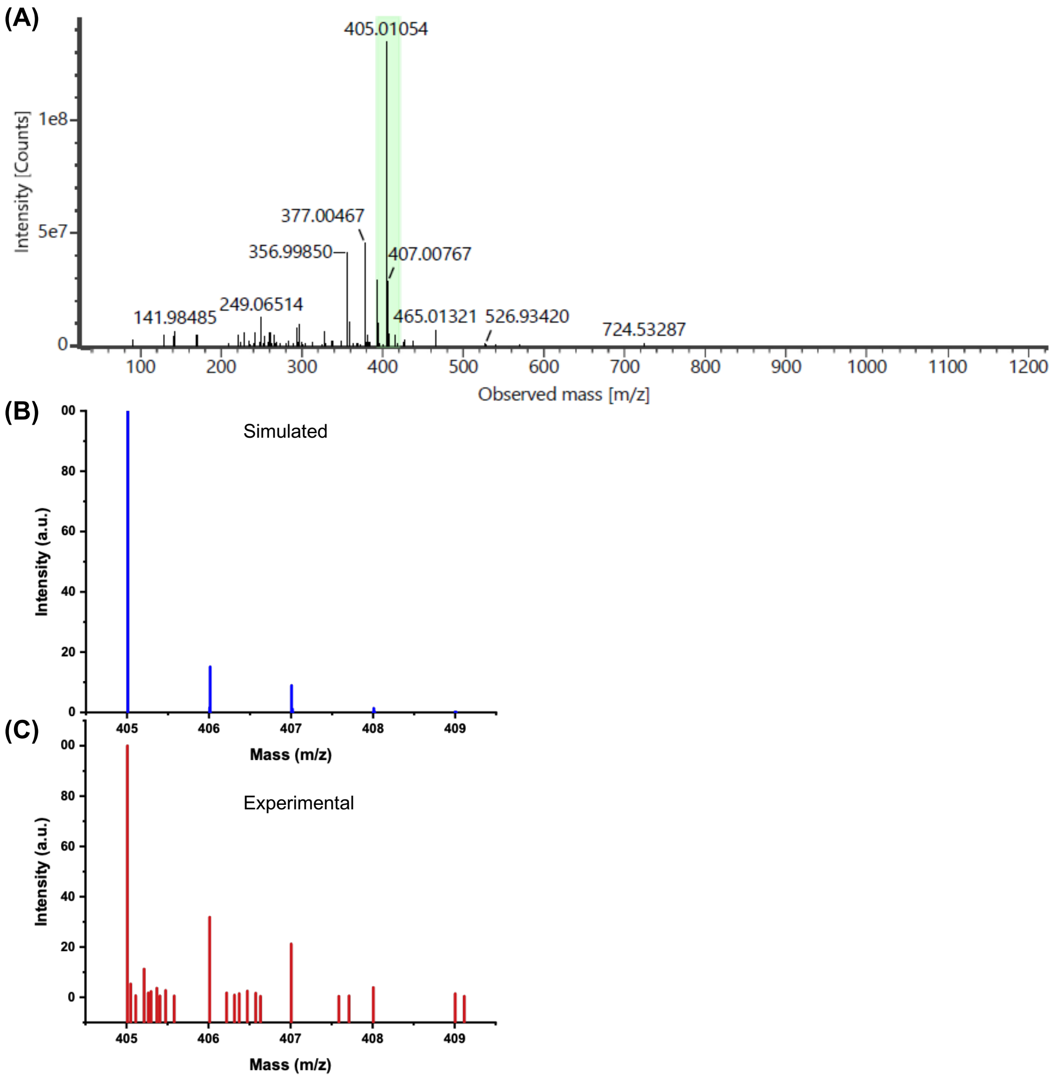

Figure S11. (A) MS spectrum of **PFPA-SS-Py.** (B) Simulated and (C) experimental MS spectra of m/z 405.

4. Synthesis of Tre-SS-Py

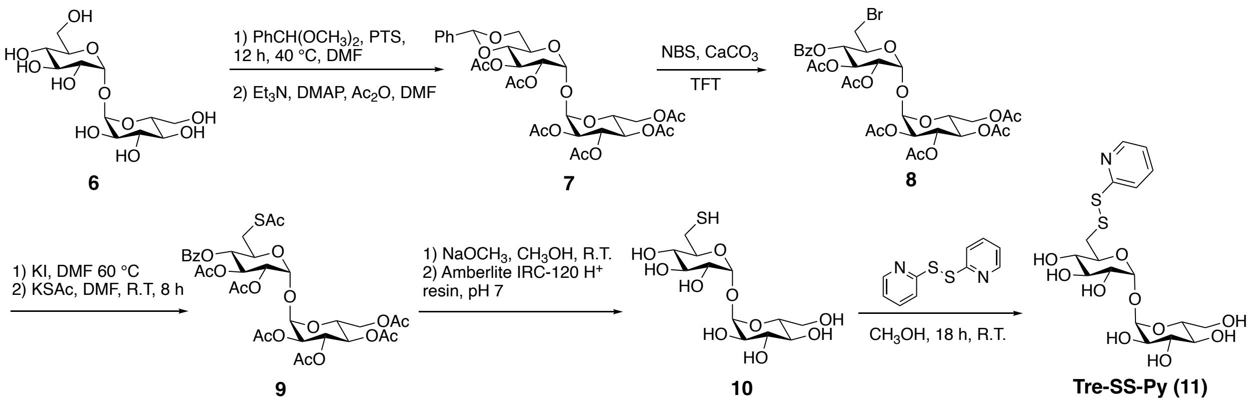

Scheme S4. Synthesis of **Tre-SS-Py**.

Synthesis of 4,6-benzylidine-2,2',3,3',6'-penta-*O*-acetyl-α,α-D-trehalose (7)[7]

D-Trehalose (**6**) (3.0 g, 8.7 mmol), benzaldehyde dimethyl acetal (1.58 mL, 10.5 mmol) and *p*-toluenesulfonic acid (0.17 g, 0.87 mmol) were dissolved in DMF (30 mL) and the solution was stirred at room temperature for 15 h. Then the reaction mixture was heated to 40 °C and stirred for another 5 h.

Acetic anhydride (10 mL, 0.11 mol), DMAP (183 mg, 1.5 mmol) and triethylamine (29 mL, 0.21 mol) were added to the above solution and stirred at room temperature for 16 h. Afterward, the DMF in the reaction mixture was distilled under reduced pressure at 60 °C. To the residue, distilled water (20 mL) was added, and the mixture was extracted with ethyl acetate (15 mL × 3). The organic phases were combined and washed with water (50 mL) and brine (50 mL). After drying over anhydrous Na_2SO_4 and concentrating under reduced pressure, the crude product was purified by column chromatography using hexanes/ethyl acetate (v/v 3:2) to yield compound **7** as a pale yellow solid (2.4 g, 40%). ^{1}H NMR (500 MHz, $CDCl_3$): δ (ppm) 7.44 – 7.42 (m, 2H), 7.36 – 7.35 (m, 3H), 5.61 (t, 1H), 5.50 – 5.49 (m, 2H), 5.37 (d, 1H), 5.28 (d, 1H), 5.07 – 5.05 (m, 2H), 5.01 (dd, 1H), 4.26 (dd, 1H), 4.23 (dd, 1H), 4.18 – 4.16 (m, 1H), 4.03 – 4.00 (m, 2H), 3.75 (t, 1H), 3.69 (t, 1H), 2.12 – 2.03 (18H).

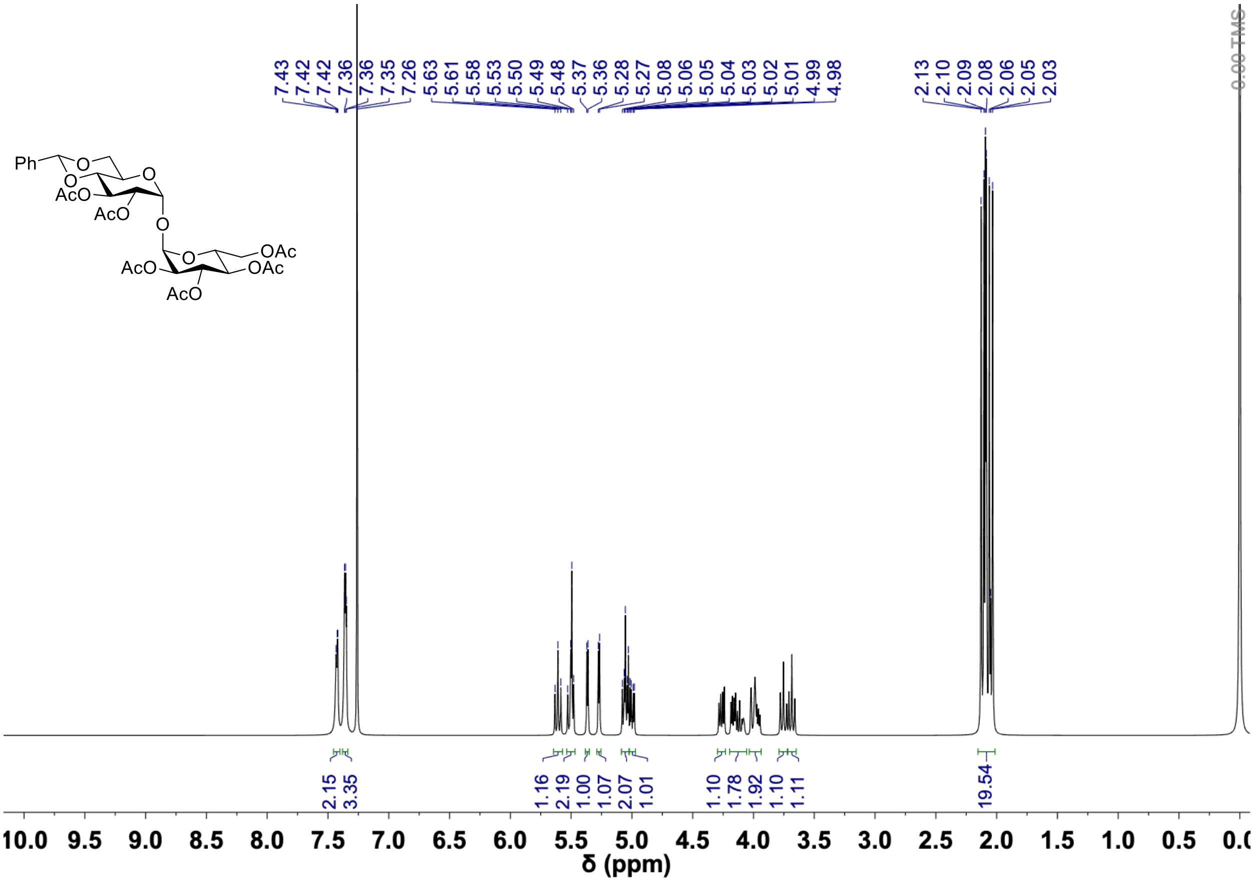

Figure S12. ^{1}H NMR spectrum of compound **7** in $CDCl_3$.

Synthesis of 4-*O*-benzoyl-6-bromo-2,2',3,3',4',6'-penta-*O*-acetyl-6-deoxy-α,α-D-trehalose (8)[7]

Compound **7** (2.0 g, 3.0 mmol) was dissolved in trifluorotoluene (100 mL) and solution was purged with Ar for 1 h while stirring. NBS (0.57 g, 3.2 mmol) and $CaCO_3$ (0.32 mg, 3.2 mmol) were added, and the reaction mixture was stirred at 77 °C for 5 h. Then the solvent was evaporated under reduced pressure. The crude product was purified by column chromatography using hexanes/ethyl acetate (v/v 3:2) to yield compound **8** as a white solid (2.1 g, 90%). ^1H NMR (500 MHz, $CDCl_3$): δ (ppm) 8.04 (dd, 2H), 7.63 (tt, 1H), 7.48 (t, 2H), 5.72 (t, 1H), 5.55 (t, 1H), 5.41 – 5.38 (m, 2H), 5.22 – 5.19 (m, 2H), 5.13 – 5.08 (m, 2H), 4.25 – 4.22 (m, 2H), 4.07 – 4.05 (m, 2H), 3.42 – 3.39(dd, 2H), 2.13 – 1.94 (18H).

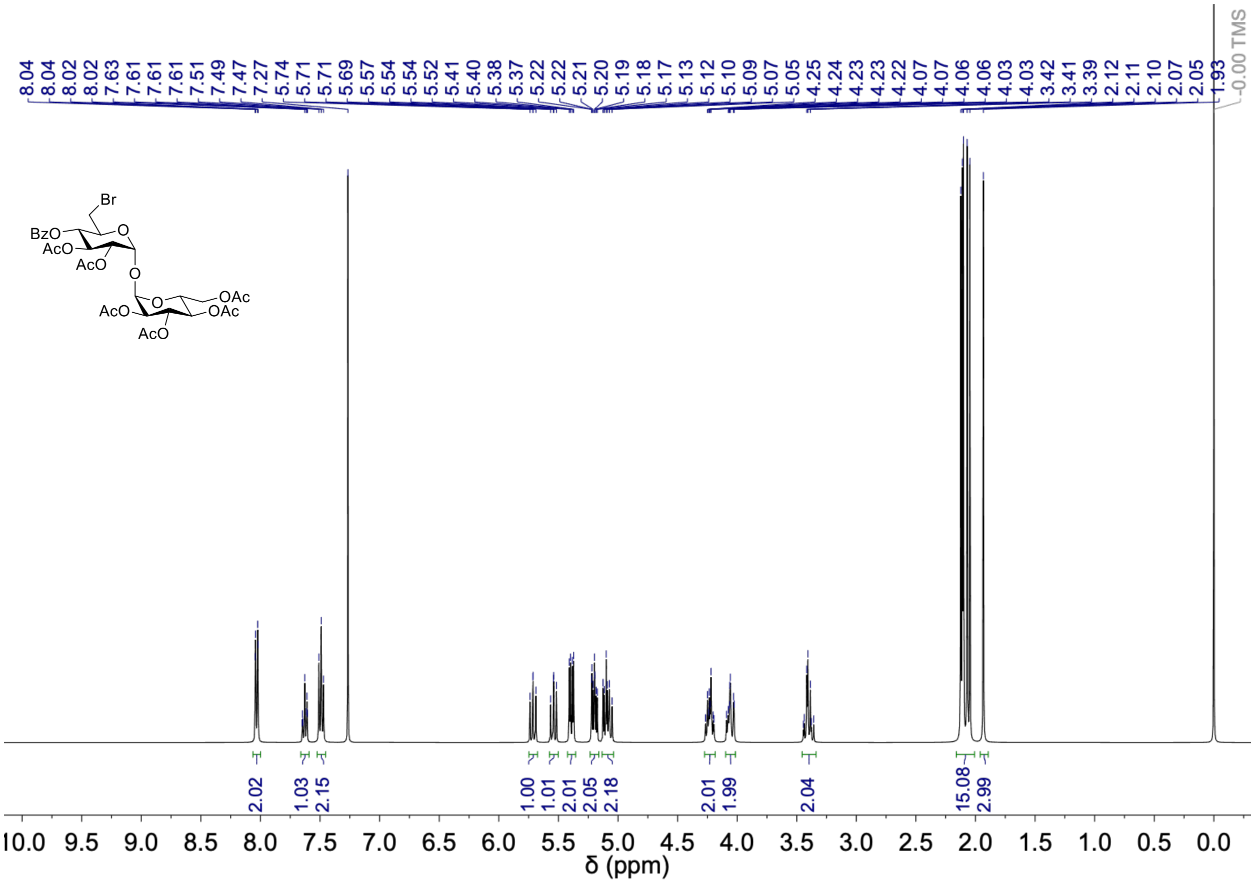

Figure S13. ^1H NMR spectrum of compound **8** in $CDCl_3$

Synthesis of 4-*O*-benzoyl-6-thioethanoyl-2,2',3,3',4',6'-penta-*O*-acetyl-6-deoxy-α,α-D-trehalose (9)[7]

Compound **8** (1.00 g, 1.3 mmol) was dissolved in DMF (20 mL). Potassium iodide (1.10 g, 6.6 mmol) was added and the reaction mixture was stirred at 60 °C for 5 h. The solvent was evaporated under reduced pressure and the crude was extracted into dichloromethane (40 mL x 3). To the crude product dissolved in DMF (30 mL), potassium thioacetate (450 mg, 3.9 mmol) was added. The mixture was stirred at room temperature for 8 h under a blanket of N_2. The product mixture was extracted with dichloromethane (40 mL x 3) and concentrated under reduced pressure. Purification by flash column chromatography (2:1 v /v hexanes:ethyl acetate) gave **9** as an off-white powder (657 mg, 66%) ^{1}H NMR (500 MHz, $CDCl_3$): δ (ppm) 8.03 (d, 2H, *o*-benzyl C-H), 7.60 (t, 1H), 7.47 (t, 2H), 5.63 (t, 1H), 5.48 (t, 1H), 5.34 (d, 2H), 5.20 (t, 1H), 5.10 - 4.98 (m 3H), 4.14 (dd, 1H), 4.04 (d, 1H), 3.95 - 3.89 (m, 2H), 3.26 (dd, 1H), 2.87 (dd, 1H), 2.28 (s, 3H), 2.07 - 1.90 (m, 18H).

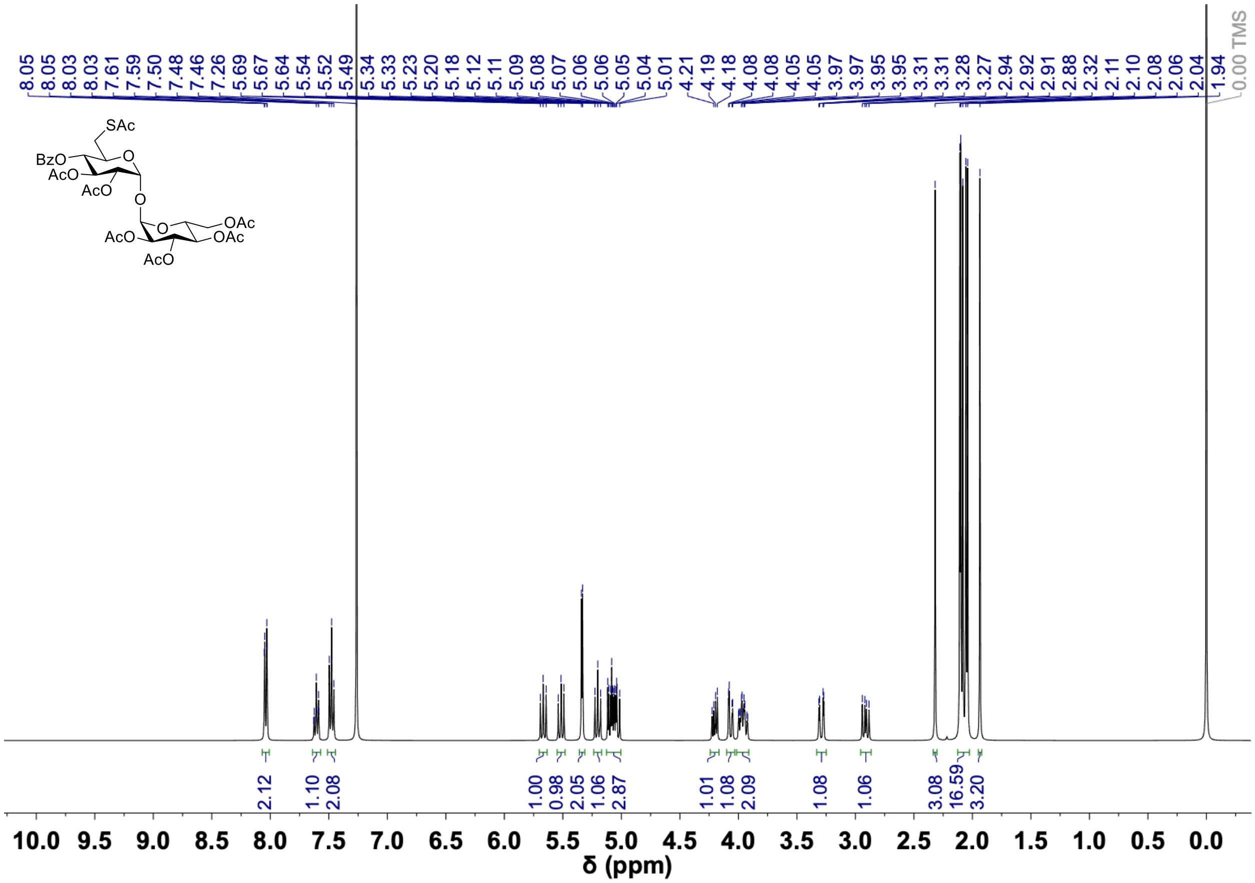

Figure S14. ^{1}H NMR spectrum of compound **9** in $CDCl_3$

Synthesis of 6-mercapto-6-deoxy-α,α-D-trehalose (10)[7]

A solution of sodium methoxide in methanol (25.0 wt%, 133 µL, 0.6 mmol) was added dropwise to compound **9** (400 mg, 0.5 mmol) in methanol (20 mL). The reaction was stirred at room temperature for 6 h. The pH of the reaction was adjusted to 7 using Amberlite® IRC-120 H^+ resin beads. After filtering the mixture, D,L-dithiothreitol (DTT) (163 mg, 1.1 mmol) was added and the reaction was allowed to stir at room temperature for 8 h. After removing the solvent, the crude was purified by column chromatography (1:3 v /v chloroform:methanol) to yield the product **10** as an off-white solid (42 mg, 22%). ^{1}H NMR (500 MHz, D_2O): δ (ppm) 5.20 (d, 1H), 5.14 (d, 1H), 3.88 - 3.67 (m, 6H), 3.60 (dt, 2H), 3.38 (t, 2H), 2.92 (dd, 1H), 2.66 (dd, 1H).

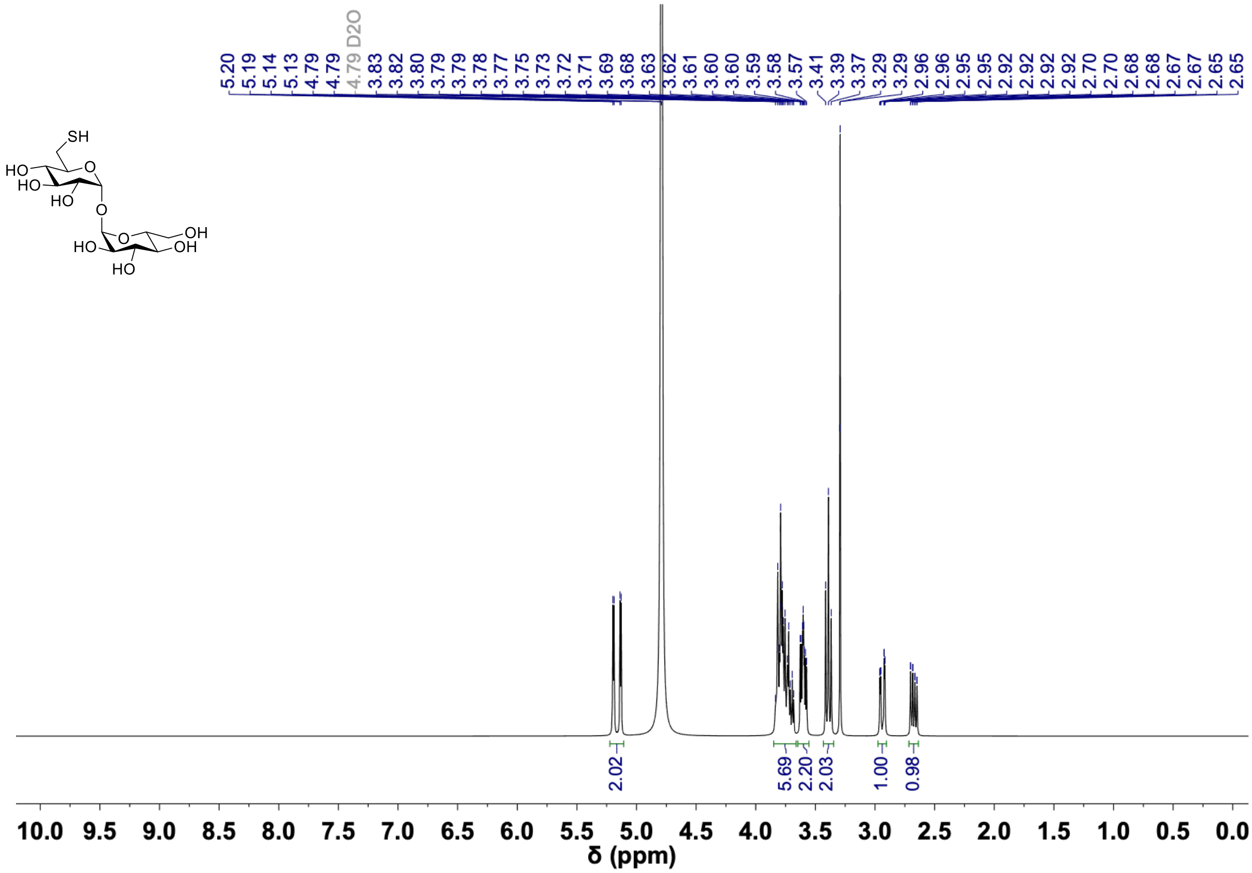

Figure S15. ^{1}H NMR spectrum of compound **10** in D_2O.

Synthesis of Tre-SS-Py

Into a solution of compound **10** (100 mg, 0.3 mmol) in methanol (20 mL) purged with Ar for 1 h was added a solution of 2,2'-dithiopyridine (61.5 mg, 0.3 mmol) in 10 mL of methanol. The solution was stirred at room temperature for 18 h. Solvent was evaporated under reduced pressure and the crude product was purified by flash column chromatography (1:3 v /v chloroform:methanol) to yield product **Tre-SS-Py** as a white solid (81 mg, 62%). ^{1}H NMR (400 MHz, methanol-d$_4$): δ (ppm) 8.35 (d, 1H), 7.86 (d, 1H), 7.79 (dt, 1H), 7.18 (t, 1H), 5.13 (d, 1H), 5.04 (d, 1H), 4.07 (dt, 1H), 3.76 (m, 4H), 3.65 (dd, 1H), 3.45 (dt, 2H), 3.25 (m, 3H), 3.02 (dd, 1H). ^{13}C NMR (400 MHz, methanol-d$_4$): δ (ppm) 161.64, 150.27, 139.26, 122.21, 121.20, 95.08, 94.75, 74.74, 74.25, 73.89, 73.17, 72.13, 71.87,62.58, 58.32, 43.65, 18.37.

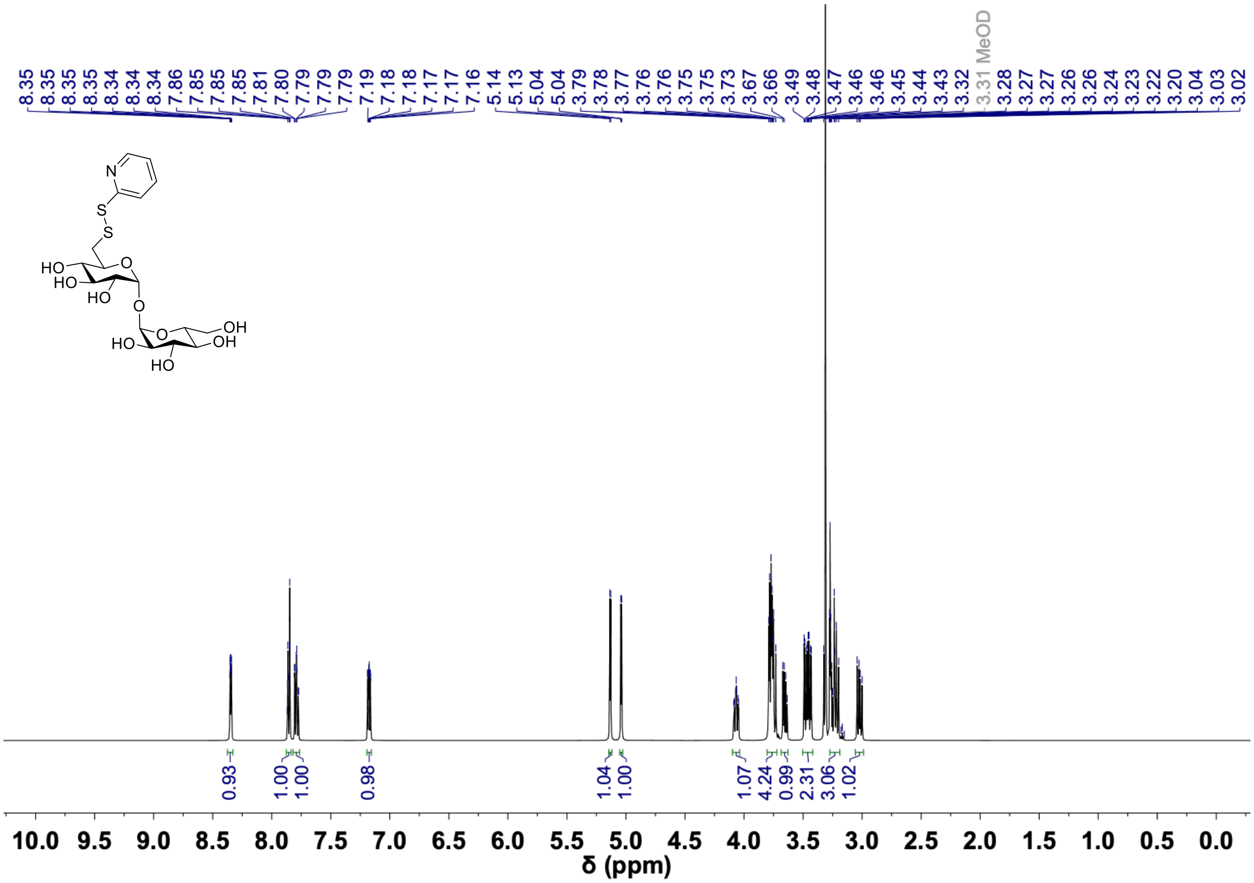

Figure S16. ^{1}H NMR spectrum of **Tre-SS-Py** in CD_3OD.

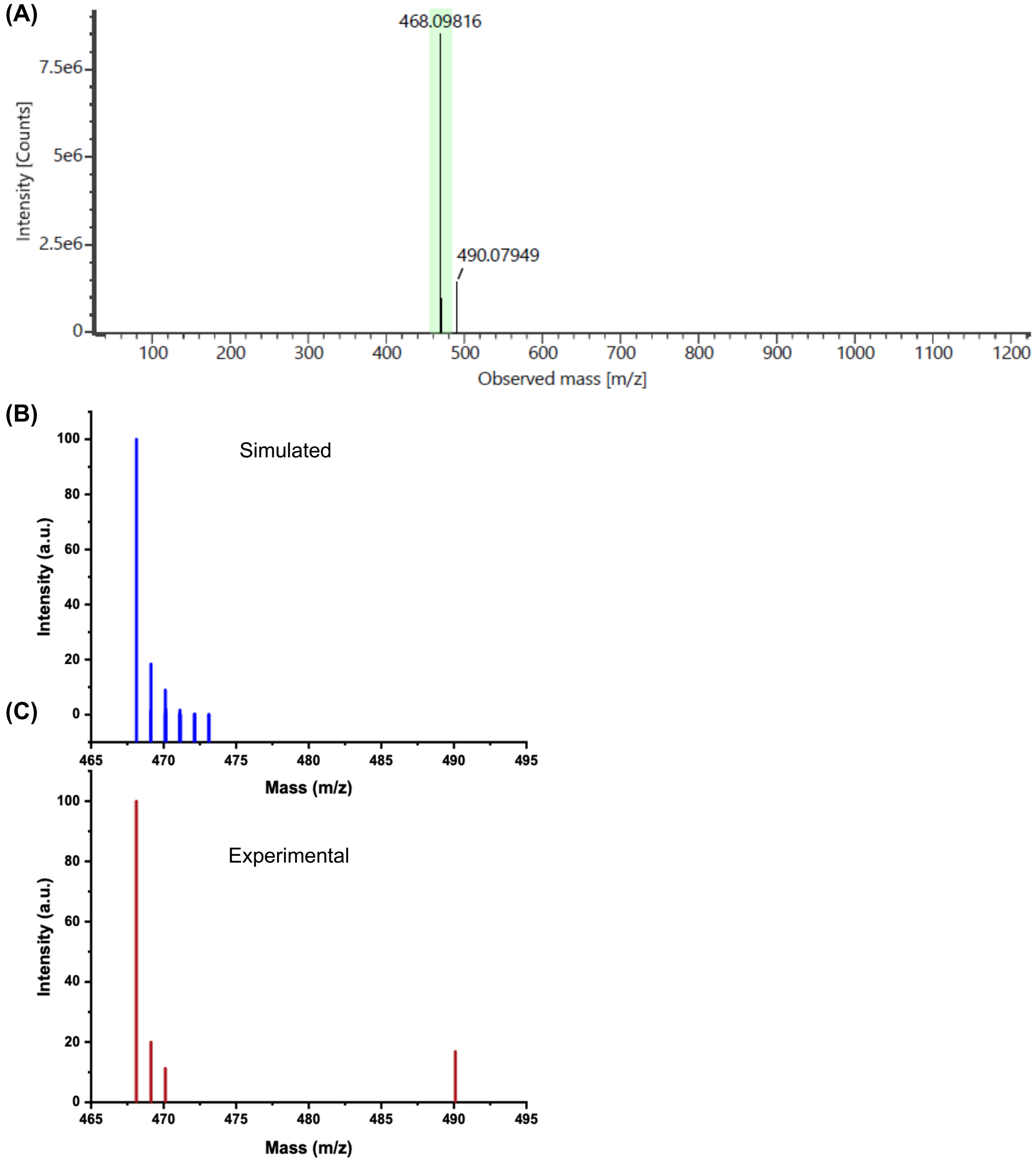

Figure S17. (A) MS spectrum of **Tre-SS-Py.** (B) Simulated and (C) experimental MS spectra of m/z 468.

5. Quantification of thiol concentration on nanoparticles using Ellman assay

Ellman assays were carried out in 0.1 M pH 8.0 phosphate buffer containing 1 mM EDTA, prepared by adding 23.3 mL of 1 M Na_2HPO_4, 1.7 mL of 1 M NaH_2PO_4, 5 mL of 0.05 M EDTA and topping up with 220 mL of water and adjusting the pH to 8.0. The Ellman reagent was prepared

by dissolving 4 mg of 5,5-dithio-bis-(2-nitrobenzoic acid) (DTNB) in 1 mL of the phosphate buffer.

(3-Mercaptopropyl)trimethoxysilane (MPTMS) was used as the calibration. Solutions of MPTMS ranging from 0.1 mM – 1.5 mM were prepared in the pH 8.0 phosphate buffer. To a set of test tubes each containing 2.5 mL of the pH 8.0 phosphate buffer, 250 µL of the MPTMS solution and 50 µL of Ellman's reagent were added. The solutions were thoroughly mixed at room temperature for 15 min. The absorbance at 412 nm was measured, and the results were plotted against the MPTMS concentration to obtain the calibration curve, **Fig. S19**.

For nanoparticle samples, 4 mg of nanoparticles dispersed in 250 µL of 0.1 M pH 8.0 phosphate buffer, 50 µL of Ellman reagent and 2.5 mL of the buffer were thoroughly mixed at room temperature for 15 min. It was then centrifuged at 10,000 rpm for 5 min to precipitate the nanoparticles. A volume of 0.3 mL of the supernatant was taken and the absorbance at 412 nm was measured. The thiol concentration was calculated by comparing the absorbance with the MPTMS calibration curve, **Fig. S19**.

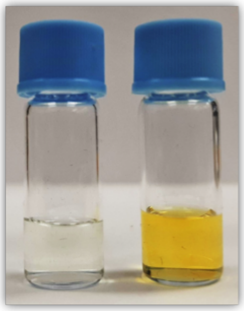

Figure S18. The Ellman reagent DTNB before (left) and after (right) the addition of MNP-SH.

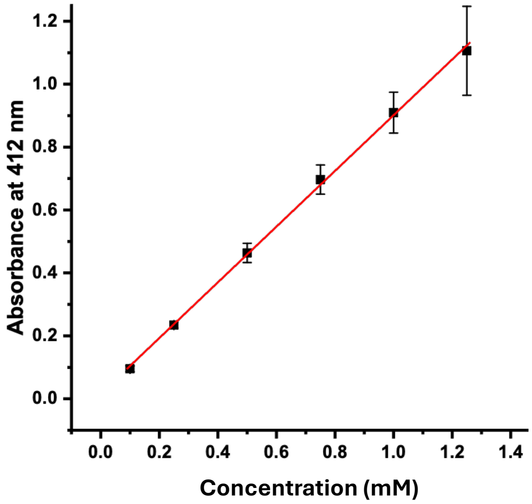

Figure S19. The Ellman calibration curve prepared from 2-(dimethylamino)ethanethiol hydrochloride. The results are the average of 3 independent experiments. R^2 = 0.9989

6. Calibration of pyridine-2-thione

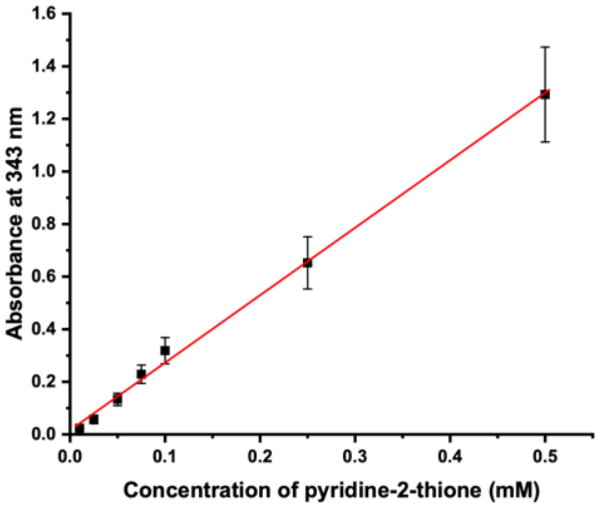

Figure S20. Absorbance at 343 nm vs. concentration of pyridine-2-thione in methanol. The results are the average of 3 independent experiments. $R^2 = 0.9971$

7. Ligand conjugation yields

Table S1. Ligand conjugation yields calculated by measuring the concentration of released pyridine-2-thione

	**First Step**[a]	**Second Step**[a]	**Overall Yield**[b]
**PFPA-MNP-Tre**			
Sample 1	86%	60%	73%
Sample 2	90%	66%	78%
Sample 3	84%	60%	72%
Ave ± SD [c]	87% ± 3%	62% ± 4%	74% ± 3%
**PFPA-MNP-OH**			
Sample 1	76%	92%	84%
Sample 2	82%	86%	84%
Sample 3	92%	82%	87%
Ave ± SD [c]	83% ± 8%	87% ± 5%	85% ± 2%

[a] See **Scheme 2**. First step: MNP-SH + Tre-SS-Py → HS-MNP-Tre. Yield is based on the concentration of the limiting agent Tre-SS-Py. Second step: HS-MNP-Tre + PFPA-SS-Py → PFPA-MNP-Tre. Yield is based on the concentration of the limiting agent PFPA-SS-Py.
[b] MNP-SH → PFPA-MNP-Tre
[c] Ave ± SD: Average ± standard deviation of the three samples.

Table S2. Thiol concentration and zeta potential of nanoparticle samples, and ligand conjugation yields calculated from the thiol concentrations on nanoparticles

	**[Thiol] (mmol/mg particle)[a]**		**Zeta potential (mV)**
**MNP**			
Sample 1	8.80×10^{-6}		ND [b]
Sample 2	9.42×10^{-6}		ND [b]
Sample 3	9.89×10^{-6}		ND [b]
**MNP-SH**			
Sample 1	1.19×10^{-4}		-79.3
Sample 2	2.76×10^{-4}		-93.2
Sample 3	1.62×10^{-4}		-82.0
Ave ± SD	$(1.86 \pm 0.81) \times 10^{-4}$		-(84.8 ± 7.3)
**PFPA-MNP-Tre**		Ligand conjugation yield%	
Sample 1	1.57×10^{-5}	87%	-8.4
Sample 2	2.30×10^{-5}	92%	-9.4
Sample 3	3.80×10^{-5}	77%	-10.3
Ave ± SD	$(2.56 \pm 1.14) \times 10^{-5}$	85% ± 8%	-(9.4 ± 1.0)
**PFPA-MNP-OH**		Ligand conjugation yield%	
Sample 1	1.61×10^{-5}	86%	-3.9
Sample 2	1.20×10^{-5}	96%	-3.7
Sample 3	4.68×10^{-6}	97%	-2.2
Ave ± SD	$(1.09 \pm 0.58) \times 10^{-5}$	93% ± 6%	-(3.3 ± 0.9)

[a] Measured by the Ellman assay.
[b] Not determined due to poor solubility in PBS.

8. TEM image and FT-IR spectrum of PFPA-MNP-Tre

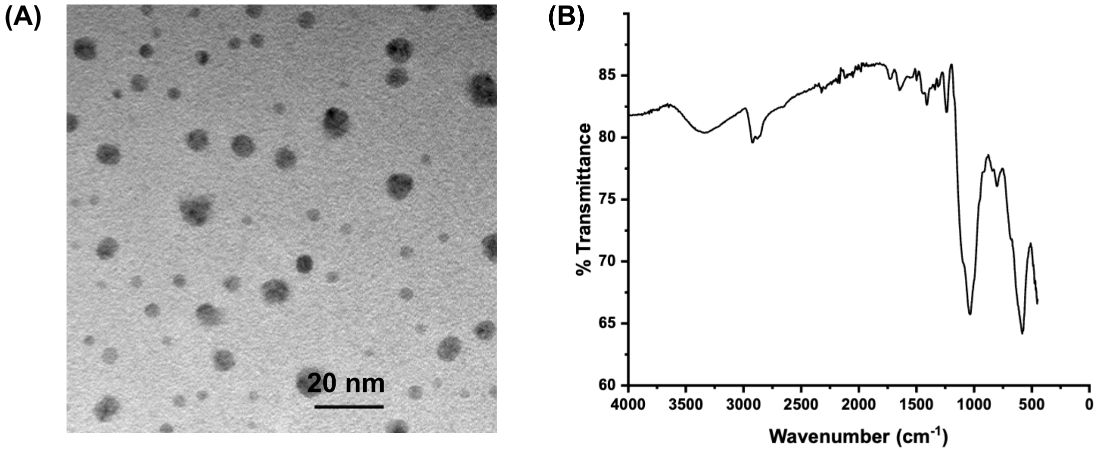

Figure S21. (A) TEM image of PFPA-MNP-Tre. The particle dimeter was calculated to be 9.8 ± 2.7 nm by averaging over 50 particles using ImageJ. (B) FT-IR spectrum of PFPA-MNP-Tre.

9. TEM image and FT-IR spectrum of PFPA-MNP-OH

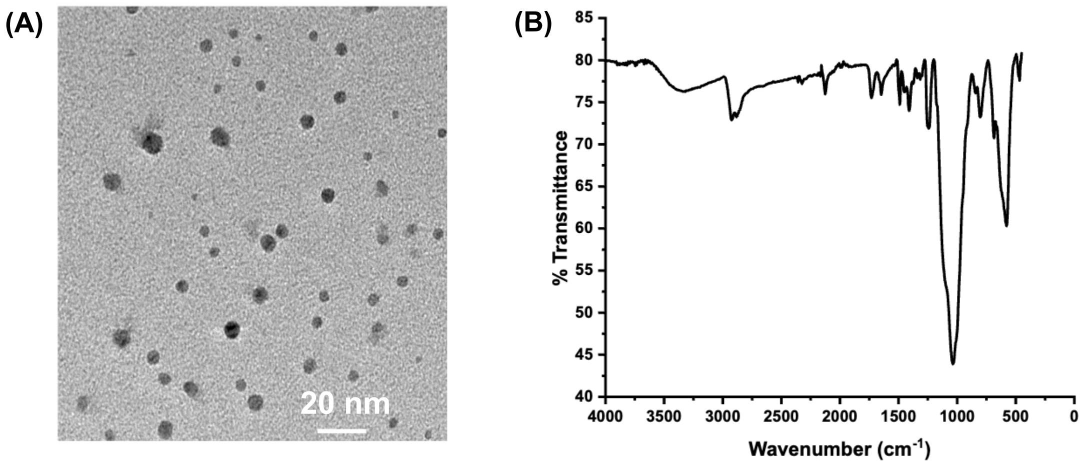

Figure S22. (A) TEM image of PFPA-MNP-OH. The particle dimeters were calculated using the ImageJ software to be 7.0 ± 1.5 nm by averaging over 50 particles. (B) FT-IR spectrum of PFPA-MNP-OH.

10. TGA data

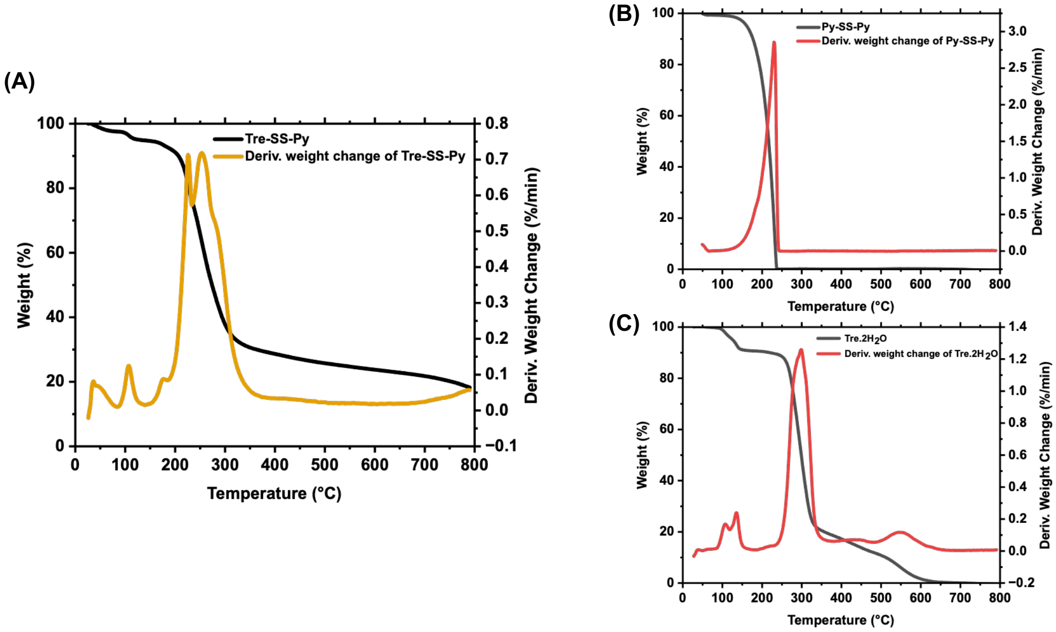

Figure S23. TGA and DTG curves of (A) **Tre-SS-Py**, (B) Py-SS-Py, (C) trehalose dihydrate. By comparing the results in (B) and (C), it can be concluded that the decomposition of Tre-SS-Py at ~225 °C was due to the loss of S-Py group (*cf* B), while decomposition at below ~125 °C and at ~250 °C and onwards were due to the decomposition of trehalose (*cf* C).

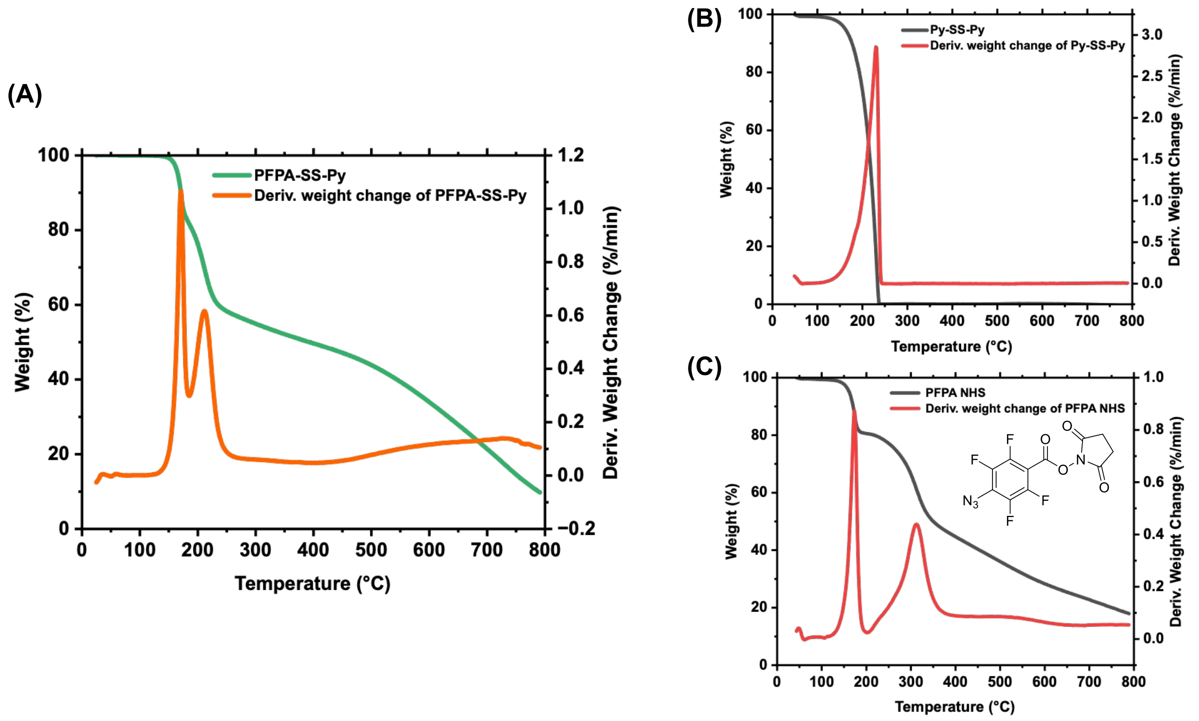

Figure S24. TGA and DTG curves of (A) **PFPA-SS-Py**, (B) Py-SS-Py, (C) PFPA NHS ester. By comparing the results in (B) and (C), it can be concluded that the decomposition of PFPA-SS-Py at ~175 °C was due to the loss of S-Py group (*cf* B), while decomposition at ~225 °C and onwards were due to the decomposition of PFPA (*cf* C). PFPA-SS-Py and PFPA NHS ester do not completely decompose. This can be contributed to self-reaction/crosslinking of PFPA in the solid state at neat conditions.[8]

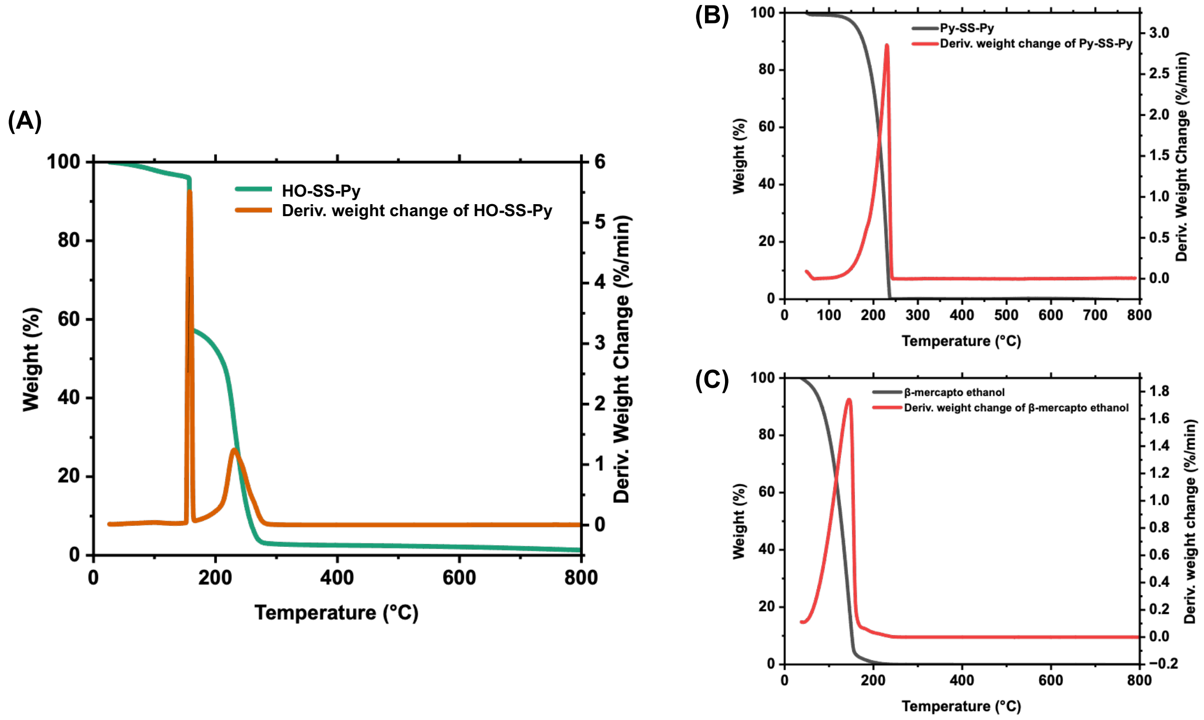

Figure S25. TGA and DTG curves of (A) **HO-SS-Py**, (B) Py-SS-Py, (C) 2-mercaptoethanol. By comparing the results in (B) and (C), it can be concluded that the decomposition of Py-SS-OH at ~155 °C was due to the loss of S-Py group (*cf* B), while decomposition at ~235 °C was due to the decomposition of mercaptoethanol group (*cf* C).

11. Calculation of ligand density on PFPA-MNP-Tre and PFPA-MNP-OH

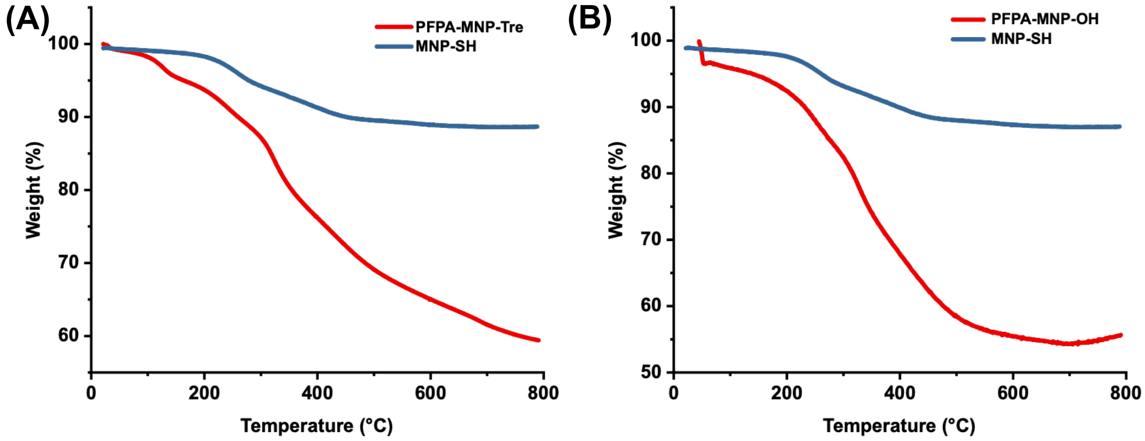

Figure S26. (A) TGA curves of PFPA-MNP-Tre and MNP-SH. (B) TGA curves of PFPA-MNP-OH and MNP-SH.

To calculate the ligand density on PFPA-MNP-Tre or PFPA-MNP-Tre, the following formula is used,[9]

$$\frac{Wx}{3Wy}\rho r \quad \text{(eq. S1)}$$

For PFPA-MNP-Tre:

W_x = weight% difference between PFPA-MNP-Tre and MNP-SH at 800 °C = 29%

W_y = weight% of sample at 800 °C = 60%

ρ = density of MNPs, which is 4.8 g/cm^3 assuming the same density as Fe_3O_4

r = radius of PFPA-MNP-Tre, which is 4.9 nm according to TEM (**Fig. S21A**)

The total ligand density is calculated as 3.8×10^{-15} µg/nm^2.

For PFPA-MNP-OH:

The total ligand density on PFPA-MNP-OH was calculated according to eq. S1:

W_x = weight% difference between PFPA-MNP-OH and MNP-SH at 800 °C = 32%

W_y = weight% of sample at 800 °C = 55%

ρ = density of MNPs, which is 4.8 g/cm^3 assuming the same density as Fe_3O_4

r = radius of PFPA-MNP-OH, which is 3.5 nm according to TEM (**Fig. S22A**)

The total ligand density is 3.3×10^{-15} µg/nm^2.

12. Optimization of conditions for capturing *M. smegmatis* proteins with PFPA-MNP-Tre in live bacteria

Bacterial concentration. *M. smegmatis* mc^2155 at different concentrations (OD_{600}: 0.07, 0.2, 0.5, 0.7) were tested to determine which concentration would give sufficient amount of proteins for SDS-PAGE analysis. Results in **Fig. S27** revealed that the protein bands were too light for OD_{600} 0.2 and 0.07. At 0.7, proteins were visible without the need for concentrating with cold acetone, whereas similar results were obtained with 0.5 after concentrating the proteins with cold acetone. Since the desired results can be obtained with 0.5, bacterial concentration of 0.5 OD_{600} was chosen for subsequent studies.

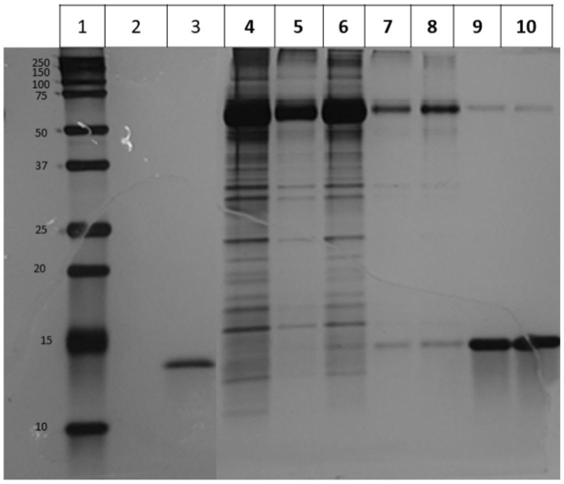

1. Molecular weight ladder
2. Blank
3. Lysozyme
4. OD_{600} 0.7
5. OD_{600} 0.5
6. OD_{600} 0.5 (after precipitation with cold acetone)
7. OD_{600} 0.2
8. OD_{600} 0.2 (after precipitation with cold acetone)
9. OD_{600} 0.07
10. OD_{600} 0.07 (after precipitation with cold acetone)

Figure S27. Silver-stained SDS-PAGE gel image of captured proteins obtained from different concentrations of *M. smegmatis* cell lysate. The band seen around 66 kD_a in lanes 4-10 corresponded to albumin which was added to the culture medium.

Concentration of PFPA-MNP-Tre. Different concentrations of PFPA-MNP-Tre (0.1, 0.2, 0.5, 1 mg/mL) dispersed in PBS (pH 7.4) were prepared. A volume of 200 µL from each PFPA-MNP-Tre concentration was added to 200 µL of 0.5 OD_{600} *M. smegmatis* in a 24-well plate and incubated at 37 °C for 24 h. After incubation, samples were subjected to irradiation, cell lysis, and disulfide cleavage as described in the Method section in the main text. The isolated proteins were run on SDS PAGE and visualized after silver staining. As shown in **Fig. S28**, a band was obtained in the region of 25-30 kD_a, which will be referred to as the protein of interest (POI) on the following sections. The POI band obtained from 0.1 and 0.2 mg/mL PFPA-MNP-Tre (lane 9 & 10) were faint. Concentrations of 0.5 and 1 mg/mL (lane 7 & 8) did not show significant differences in the band intensity. Therefore, concentration of 0.5 mg/mL was chosen for subsequent studies.

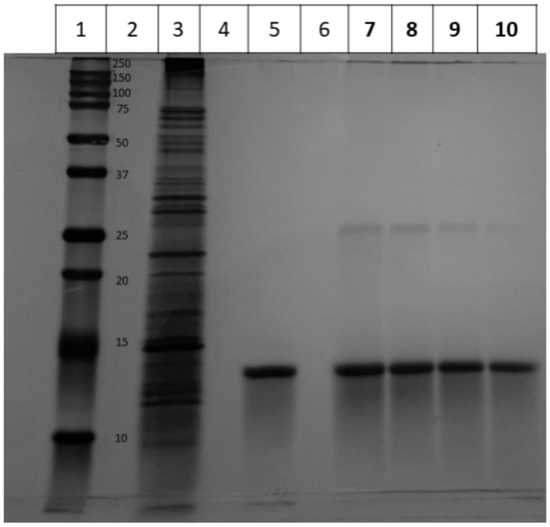

1. Molecular weight ladder
3. *M. smegmatis* cell lysate
5. Lysozyme
7. 1 mg/mL PFPA-MNP-Tre
8. 0.5 mg/mL PFPA-MNP-Tre
9. 0.2 mg/mL PFPA-MNP-Tre
10. 0.1 mg/mL PFPA-MNP-Tre

Figure S28. Silver stained SDS-PAGE gel image of isolated proteins after treating *M. smegmatis* with different concentrations of PFPA-MNP-Tre at 37 °C for 24 h.

Incubation time. A volume of 250 µL of 0.5 mg/mL PFPA-MNP-Tre was incubated with 250 µL of 0.5 OD_{600} *M. smegmatis* at 37 °C in the dark for 4 h, 6 h, 18 h and 24 h, respectively. After incubation, samples were subjected to irradiation, cell lysis, and disulfide cleavage as described in the Method section in the main text. The isolated proteins were run on SDS PAGE and visualized after silver staining. According to results in **Fig. S29**, 24 h gave the highest intensity of the POI band.

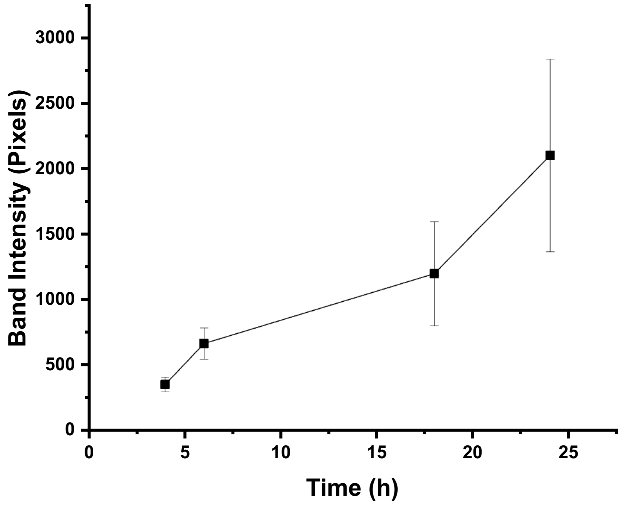

Figure S29. The POI band intensity vs. incubation time. Band intensities were measured in pixels using ImageJ. Data were the average of two independent trials.

Irradiation time. PFPA-SS-Py was used to test the irradiation time needed to complete the photochemical reaction of PFPA by monitoring the intensity of the azide absorption by FTIR. A solution of PFPA-SS-Py (50 µg/mL) was prepared in methanol in a 20 mL glass vial. Three different samples of the same solution were covered with a 280 nm long-pass optical filter and was irradiated using a 450-W HANOVIA medium-pressure Hg lamp (intensity was 2.5 mW/cm^2 at 365 nm at the location of the sample) for 10, 20 and 30 min, respectively. The solvent was removed under reduced pressure, and FT-IR spectra were recorded. The FTIR spectra of the product obtained after 10 and 20 min irradiation still showed the azide peak at 2127 cm^{-1}. After 30 min, the peak corresponding to azide (indicated with an arrow) disappeared as shown in **Fig. S30**. Therefore, 30 min irradiation time was chosen for the subsequent studies.

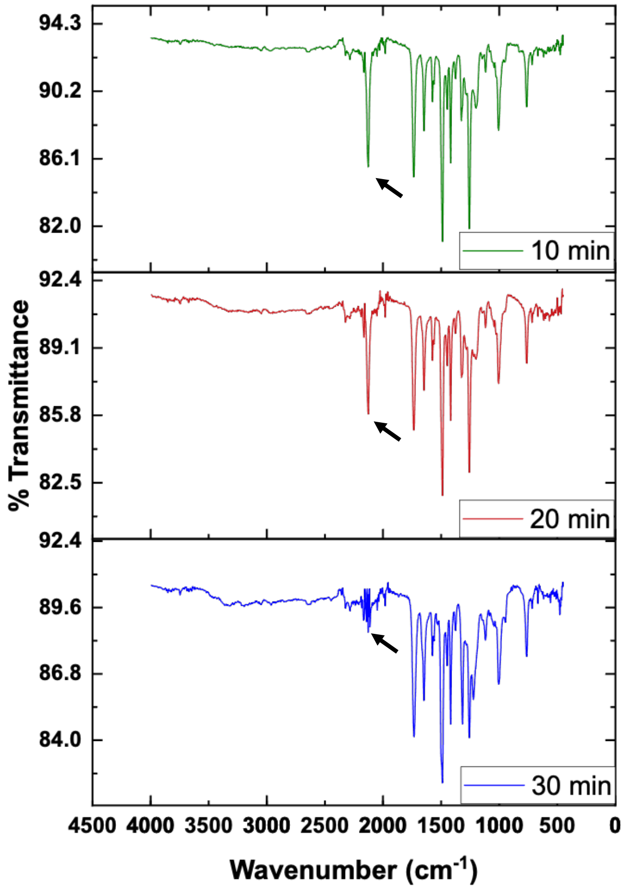

Figure S30. FT-IR spectra of PFPA-SS-Py after irradiation for 10 min (top), 20 min (middle) or 30 min (bottom).

13. Identification of isolated proteins by LC-MS/MS

Resolved protein bands were cut from the gel and were subjected to LC-MS/MS followed by database search to identify the proteins. Protein sequence analysis by LC-MS/MS was performed by Taplin Biological Mass Spectrometry Facility following the protocol below.

Excised gel bands were cut into approximately 1 mm^3 pieces. Gel pieces were then subjected to a modified in-gel trypsin digestion procedure.[10] Gel pieces were washed and dehydrated with acetonitrile for 10 min followed by removal of acetonitrile. Pieces were then completely dried in a speed-vac. Rehydration of the gel pieces was done with 50 mM ammonium bicarbonate solution containing 12.5 ng/µL modified sequencing-grade trypsin (Promega, Madison, WI) at 4 °C. After 45 min, the excess trypsin solution was removed and replaced with 50 mM ammonium bicarbonate solution to just cover the gel pieces. Samples

were then placed in a 37 °C room overnight. Peptides were later extracted by removing the ammonium bicarbonate solution, followed by one wash with a solution containing 50% acetonitrile and 1% formic acid. The extracts were then dried in a speed-vac (~1 h). The samples were stored at 4 °C until analysis.

On the day of analysis, the samples were reconstituted in 5–10 µL of HPLC solvent A (2.5% acetonitrile, 0.1% formic acid). A nano-scale reverse-phase HPLC capillary column was created by packing 2.6 µm C18 spherical silica beads into a fused silica capillary (100 µm inner diameter × ~30 cm length) with a flame-drawn tip.[11] After equilibrating the column, each sample was loaded via a Famos auto sampler (LC Packings, San Francisco CA) onto the column. A gradient was formed, and peptides were eluted with increasing concentrations of solvent B (97.5% acetonitrile, 0.1% formic acid).

As peptides eluted, they were subjected to electrospray ionization and then entered into an LTQ Orbitrap Velos Pro ion-trap mass spectrometer (Thermo Fisher Scientific, Waltham, MA). Peptides were detected, isolated, and fragmented to produce a tandem mass spectrum of specific fragment ions for each peptide. Peptide sequences (and hence protein identity) were determined by matching protein databases with the acquired fragmentation pattern by Sequest software (Thermo Fisher Scientific, Waltham, MA).[12] All databases include a reversed version of all the sequences and the data were filtered to fall between one and two percent peptide false discovery rate.

Table S3. LC-MS/MS analysis of POI protein band.[a]

**Gene Symbol**	**XCorr**[b]	**ΔCorr**[c]	**# Ions**[d]	**Protein**	**MW (kDa)**	**Location**	**Function and/or process involved**
MSMEG_6189	4.709	0.541	21/30	Transcriptional regulator, Crp/Fnr family protein	24.76	Cytosol	Protein transcription[13]
	2.203	0.274	10/12				
	2.167	0.457	12/14				
	4.119	0.358	18/32				
	2.095	0.429	11/16				
	1.660	0.206	11/12				
prrA	4.610	0.610	21/30	DNA-binding response regulator PrrA	25.33	Cytosol	Regulation of DNA-templated transcription[14]
	4.527	0.635	21/32				
MSMEG_2079	4.637	0.674	23/28	Alcohol dehydrogenase	33.85	NA[e]	NADPH activity[15, 16]
	2.857	0.408	16/18				
gapA	3.185	0.653	15/32	Glyceraldehyde-3-phosphate dehydrogenase (GAPDH)	35.93	Cytoplasm	Catalyzes oxidative phosphorylation of glyceraldehyde 3-phosphate to 1,3-bisphosphoglycerate[17]
	2.969	0.338	16/18				
rpsE	3.311	0.663	21/26	Small ribosomal subunit protein uS5	21.90	Cytoplasm	Protein translation[18]
	2.487	0.318	19/44				
MSMEG_2941	3.297	0.661	18/26	NAD dependent epimerase/dehydratase family protein	22.18	NA	NA[19]
	2.245	0.128	21/72				
trpA	3.415	0.518	27/64	Tryptophan synthase alpha chain	27.06	Cytosol	Amino acid biosynthesis[20]
	1.722	0.329	13/26				
MSMEG_5183	2.796	0.403	12/24	3-Hydroxyacyl-CoA dehydrogenase	26.29	Cytosol	Oxidoreductase activity[21]
	1.895	0.033	11/22				

[a] Results were from two sets of independent experiments. The first sample was from 3 gel bands, and the second sample was from 12 gel bands. In the first sample, only *MSMEG_6189, rpsE,* and *gapA* were detected, with *MSMEG_6189* being the most abundant followed by *rpsE*, and then *gapA*.

[b] XCorr (cross correlation) is a score that evaluates the quality of the spectrum and the predicted fragmentation pattern of the peptide. The higher the value the better the quality of the spectrum. The values are not normalized.

[c] ΔCorr (delta correlation) measures the confidence in the first peptide match versus second possible match in the database. A ΔCorr of 0.1 or greater indicates that this peptide is significantly better than the next possible match.

[d] # Ions lists the number of ions that are believed to be matched versus the number of predicted ions.

When the scores are not perfect, the best indicator to give confidence in a protein match is the number of peptides matched that have high XCorr and ΔCorr values.

[e] NA: Not available

14. Determination of bacteria viability

Bacteria viability was determined by colony counting by growing bacteria on Middlebrook 7H10 agar plates, prepared as follows. Middlebrook 7H10 agar base (19.47 g, Sigma) and glycerol (5 mL) were topped up to 1 L with Milli-Q water, sterilized by autoclaving, and cooled to 45 °C. Middlebrook OADC Growth Supplement (M0678, 20 mL) was aseptically added, quickly swirled and poured into petri dishes. The plates were dried in the hood for 15 min and stored at 4 °C until further use.

A single colony of *M. smegmatis* mc^2 155 was inoculated from a streaked LB agar plate in a culture tube in the Sauton medium at 37 °C until OD_{600} reached 0.5. Bacteria were centrifuged at 5,000 rpm for 10 min and redispersed in pH 7.4 PBS until 0.5 OD_{600}. The bacteria in a 24-well plate (200 µL) were then incubated with PFPA-MNP-Tre or PFPA-MNP-OH dispersed in PBS (200 µL, 0.5 mg/mL) at 35 °C for 24 h. Afterwards, a dilution series of the sample was prepared (10^8 to 10^3 CFU/mL) and 20 µL of each dilution was plated in Middlebrook 7H10 agar plates. Plates were incubated at 35 °C until colonies appeared. The number of colonies was counted for each dilution. The control was the PBS-dispersed bacterial culture kept at 35 °C for 24 h without the addition of PFPA-MNP-Tre or PFPA-MNP-OH.

Table S4. Viability (CFU/mL) of *M. smegmatis* mc^2 155 after treating with PFPA-MNP-Tre or PFPA-MNP-OH.

	**Trial 1**	**Trial 2**	**Average ± SD**
After treating with PFPA-MNP-Tre	1.0×10^8	3.5×10^8	$(2.3 \pm 1.8) \times 10^8$
After incubation with PFPA-MNP-OH	1.2×10^8	1.3×10^8	$(1.3 \pm 0.1) \times 10^8$
Bacteria only control	1.5×10^8	4.0×10^8	$(2.8 \pm 1.8) \times 10^8$

15. TEM imaging

M. smegmatis mc^2 155 (250 µL, 0.5 OD_{600} in PBS) was incubated with PFPA-MNP-Tre or PFPA-MNP-OH (250 µL, 0.5 mg/mL in PBS) at 35 °C for 24 h under dark while shaking at 200 rpm. A drop (5 µL) of the mixture was placed onto a Cu grid (CF200-CU, carbon film 200 mesh, Electron

Microscopy Sciences, PA), and vacuum dried overnight. TEM images were collected on a transmission electron microscope (Philips CM12) at an acceleration voltage of 120 kV.

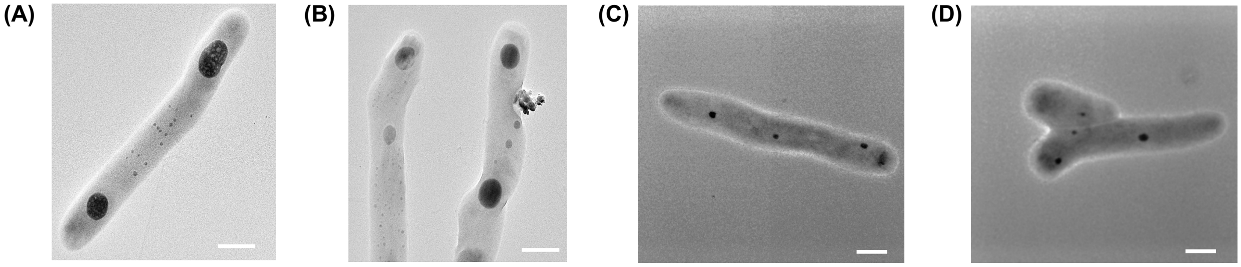

Figure S31. Additional TEM images of *M. smegmatis* after treating with (A, B) PFPA-MNP-Tre or (C, D) PFPA-MNP-OH for 24 h. Scale bars: 500 nm.

16. Competition with free trehalose

M. smegmatis mc^2 155 was cultured in the Sauton medium to 0.5 OD_{600}. A volume of 250 µL was redispersed in pH 7.4 PBS, and added to a 24-well plate, each well containing 250 µL of 0.5 mg/mL PFPA-MNP-Tre. To each well, free trehalose was added to final concentration of 0, 1 µM, 100 µM, 100 mM, 1 M respectively. The plate was incubated at 35 °C for 24 h under dark while shaking at 200 rpm. After incubation, samples were irradiated, subjected to cell lysis, purified and proteins concentrated as described in the Method section in the main text. Followed by disulfide bond cleavage, isolated proteins were run on SDS PAGE and visualized after silver staining.

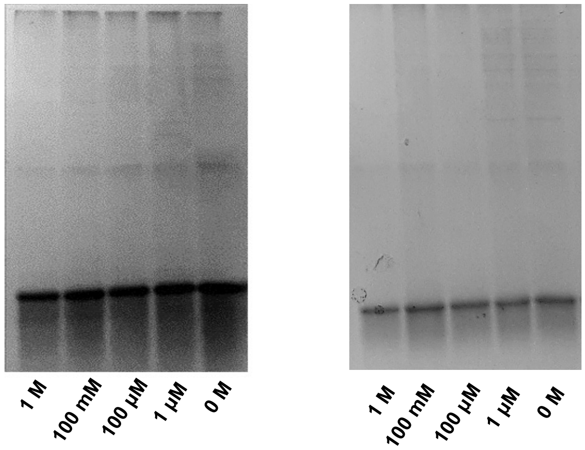

Figure S32. Silver-stained gel images of the proteins captured by PFPA-MNP-Tre in the presence of varying concentrations of free trehalose. Results in Fig. 4C are the average of these two independent trials.

17. Growth curve of *M. smegmatis* mc²155

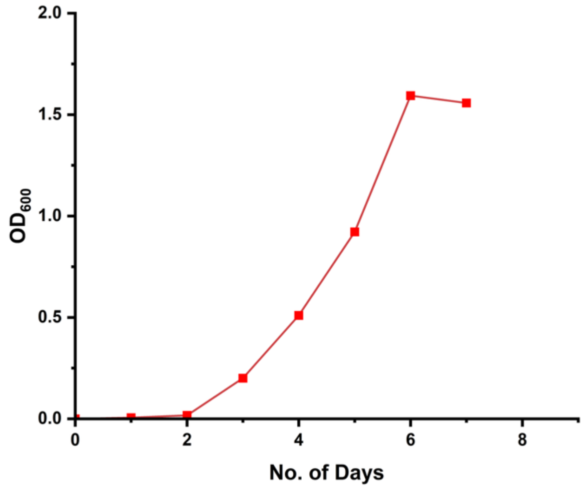

Figure S33. Growth curve of *M. smegmatis* mc²155 in Sauton's medium. The line was drawn to aid visualization.

18. References

1. Sun, S.; Zeng, H.; Robinson, D. B.; Raoux, S.; Rice, P. M.; Wang, S. X.; Li, G., Monodisperse MFe_2O_4 (M = Fe, Co, Mn) nanoparticles. *J Am Chem Soc* **2004,** *126*, 273-9.
2. Veisi, H.; Sedrpoushan, A.; Maleki, B.; Hekmati, M.; Heidari, M.; Hemmati, S., Palladium immobilized on amidoxime-functionalized magnetic Fe_3O_4 nanoparticles: a highly stable and efficient magnetically recoverable nanocatalyst for sonogashira coupling reaction. *Appl Organomet Chem* **2015,** *29*, 834-839.
3. Li, D.; Teoh, W. Y.; Gooding, J. J.; Selomulya, C.; Amal, R., Functionalization Strategies for Protease Immobilization on Magnetic Nanoparticles. *Adv Funct Mater* **2010,** *20*, 1767-1777.
4. Norberg, O.; Deng, L.; Yan, M.; Ramstrom, O., Photo-click immobilization of carbohydrates on polymeric surfaces--a quick method to functionalize surfaces for biomolecular recognition studies. *Bioconjug Chem* **2009,** *20*, 2364-70.
5. Grover, G. N.; Lee, J.; Matsumoto, N. M.; Maynard, H. D., Aminooxy and Pyridyl Disulfide Telechelic Poly(Polyethylene Glycol Acrylate) by RAFT Polymerization. *Macromolecules* **2012,** *45*, 4858-4965.
6. Zhou, J.; Hao, N.; De Zoyza, T.; Yan, M.; Ramstrom, O., Lectin-gated, mesoporous, photofunctionalized glyconanoparticles for glutathione-responsive drug delivery. *Chem Commun (Camb)* **2015,** *51*, 9833-6.
7. Wu, B.; Yang, X.; Yan, M., Synthesis and Structure-Activity Relationship Study of Antimicrobial Auranofin against ESKAPE Pathogens. *J Med Chem* **2019,** *62*, 7751-7768.
8. Liu, L. H.; Yan, M., Perfluorophenyl azides: new applications in surface functionalization and nanomaterial synthesis. *Acc Chem Res* **2010,** *43*, 1434-43.
9. Jayawardana, K. W.; Wijesundera, S. A.; Yan, M., Aggregation-based detection of M. smegmatis using D-arabinose-functionalized fluorescent silica nanoparticles. *Chem Commun (Camb)* **2015,** *51*, 15964-6.
10. Shevchenko, A.; Wilm, M.; Vorm, O.; Mann, M., Mass spectrometric sequencing of proteins silver-stained polyacrylamide gels. *Anal Chem* **1996,** *68*, 850-8.
11. Peng, J.; Gygi, S. P., Proteomics: the move to mixtures. *J Mass Spectrom* **2001,** *36*, 1083-91.

12. Eng, J. K.; McCormack, A. L.; Yates, J. R., An approach to correlate tandem mass spectral data of peptides with amino acid sequences in a protein database. *J Am Soc Mass Spectrom* **1994,** *5*, 976-89.
13. UniProt A0R5H1 · A0R5H1_MYCS2. https://www.uniprot.org/uniprotkb/A0R5H1/entry (accessed May 5, 2024).
14. UniProt A0R407 · A0R407_MYCS2. https://www.uniprot.org/uniprotkb/A0R407/entry (accessed May 10, 2024).
15. UniProt A0QU52 · A0QU52_MYCS2. https://www.uniprot.org/uniprotkb/A0QU52/entry (accessed May 10, 2024).
16. Titgemeyer, F.; Amon, J.; Parche, S.; Mahfoud, M.; Bail, J.; Schlicht, M.; Rehm, N.; Hillmann, D.; Stephan, J.; Walter, B.; Burkovski, A.; Niederweis, M., A Genomic View of Sugar Transport in Mycobacterium smegmatis and Mycobacterium tuberculosis. *J Bacteriol* **2007,** *189*, 5903-5915.
17. UniProt A0QWW2 · G3P_MYCS2. https://www.uniprot.org/uniprotkb/A0QWW2/entry (accessed May 10, 2024).
18. UniProt A0QSG6 · RS5_MYCS2. https://www.uniprot.org/uniprotkb/A0QSG6/entry (accessed May 10, 2024).
19. UniProt A0QWH2 · A0QWH2_MYCS2. https://www.uniprot.org/uniprotkb/A0QWH2/entry (accessed May 10, 2024).
20. UniProt A0QX97 · A0QX97_MYCS2. https://www.uniprot.org/uniprotkb/A0QX97/entry (accessed May 10, 2024).
21. UniProt A0R2P1 · A0R2P1_MYCS2. https://www.uniprot.org/uniprotkb/A0R2P1/entry (accessed May 10, 2024).
